# NAP1L1 regulates BIRC2 ubiquitination modification via E3 ubiquitin ligase UBR4 and hence determines hepatocellular carcinoma progression

**DOI:** 10.1038/s41420-024-01927-2

**Published:** 2024-03-27

**Authors:** Shi-Long Zhang, Shen-Jie Zhang, Lian Li, Ye-Wei Zhang, Zhi Wang, Long Wang, Jie-Yu Lu, Teng-Xiang Chen, Shi Zuo

**Affiliations:** 1 Department of Hepatobiliary Surgery, The Affiliated Hospital of Guizhou Medical University, 550001 Guiyang, China; 2 Department of Clinical Medicine, Guizhou Medical University, 550001 Guiyang, Guizhou China; 3 Breast and Thyroid Disease Center, The Third People’s Hospital of Chengdu, Sichuan, China; 4 Department of Pathophysiology, School of Basic Medical Sciences, Guizhou Medical University, 550025 Guiyang, Guizhou China; 5 Department of Physiology, School of Basic Medical Sciences, Guizhou Medical University, 550025 Guiyang, China; 6 Transformation Engineering Research Center of Chronic Disease Diagnosis and Treatment, Guizhou Medical University, 550025 Guiyang, China; 7 Guizhou Provincial Key Laboratory of Pathogenesis and Drug Research on Common Chronic Diseases, Guizhou Medical University, 550025 Guiyang, China; 8 Affiliated Hospital of Guizhou Medical University, 550001 Guiyang, China; 9 Precision Medicine Research Institute of Guizhou, The Affiliated Hospital of Guizhou Medical University, 550001 Guiyang, China

**Keywords:** Apoptosis, Oncogenesis

## Abstract

We have previously shown that nucleosome assembly protein 1-like 1 (NAP1L1) plays an important role in the abnormal proliferation of hepatocellular carcinoma (HCC) cells. However, the effects of NAP1L1 on the malignant behaviour of HCC cells, including cell migration, invasion and apoptosis, remain unclear. Baculoviral IAP repeat-containing 2 (BIRC2) plays a key role in initiating the abnormal proliferation, apoptotic escape and multidrug resistance of HCC cells; however, the mechanisms through which its stability is regulated in HCC remain elusive. Here, we found that knockdown of NAP1L1 inhibited the proliferation of HCC cells and activated apoptotic pathways but did not remarkably affect the migratory and invasive abilities of HCC cells. In addition, knockdown of NAP1L1 did not alter the expression of BIRC2 at the transcriptional level but substantially reduced its expression at the translational level, suggesting that NAP1L1 is involved in the post-translational modification (such as ubiquitination) of BIRC2. Furthermore, BIRC2 was highly expressed in human HCC tissues and promoted the proliferation and apoptotic escape of HCC cells. Co-immunoprecipitation (Co-IP) assay and mass spectrometry revealed that NAP1L1 and BIRC2 did not bind to each other; however, ubiquitin protein ligase E3 component n-recognin 4 (UBR4) was identified as an intermediate molecule associating NAP1L1 with BIRC2. Knockdown of NAP1L1 promoted the ubiquitin-mediated degradation of BIRC2 through the ubiquitin–protein junction of UBR4, which in turn inhibited the proliferation and apoptotic escape of HCC cells and exerted anti-tumour effects. In conclusion, this study reveals a novel mechanism through which NAP1L1 regulates the ubiquitination of BIRC2 through UBR4, thereby determining the progression of HCC. Based on this mechanism, suppression of NAP1L1 may inhibit tumour progression in patients with HCC with high protein expression of NAP1L1 or BIRC2.

## Introduction

Primary liver cancer is the seventh most common tumour and the second leading cause of cancer-related death worldwide, with an annually increasing incidence [1]. Hepatocellular carcinoma (HCC) is the most common subtype of liver cancer, accounting for approximately 75% of all liver cancer cases [2]. The progression of HCC is closely related to the apoptotic escape of cancer cells, which contributes to the long-term survival and abnormal proliferation of cancer cells and is an important mechanism underlying the development of multidrug resistance [3–5]. Activating or restoring tumour cell apoptosis is an effective strategy for treating tumours [6–8]. The apoptotic escape of tumour cells involves multiple signalling pathways and epigenetic modifications, including post-translational regulation of key proteins [7, 9]. Ubiquitination in the ubiquitin–proteasome system is an important post-translational modification responsible for the degradation and turnover of apoptosis-related proteins [10, 11].

Nucleosome assembly protein 1-like 1 (NAP1L1), a member of the nucleosome assembly protein 1-like protein family, has a highly conserved central structural domain, a crystal structural domain and two N-terminal structural domains. It is distributed primarily in the cytoplasm and to a lesser extent in the nucleus [12]. Several studies have suggested that NAP1L1 is a potential pro-tumorigenic factor and is involved in the progression of malignant tumours such as HCC, colorectal cancer and breast cancer [13–16]. We have previously reported that NAP1L1 expression is upregulated in HCC tissues and is associated with a poor prognosis. Upregulated NAP1L1 increases the expression of the cell cycle protein cyclin D1 (CCND1) by recruiting heparin-binding growth factor (HDGF) and interacting with c-Jun, thereby enhancing the proliferation of HCC cells and promoting tumour progression [13]. However, we did not examine the effects of NAP1L1 on other malignant behaviours of HCC cells, such as cell migration, invasion and apoptosis. Previous studies have demonstrated that knockdown of NAP1L1 depolarises the mitochondrial membrane potential, which in turn mediates tumour cell apoptosis [17, 18]. Therefore, elucidating the effects of NAP1L1 on different malignant characteristics of HCC cells may provide insights into the physiological functions of NAP1L1.

Baculoviral IAP repeat-containing 2 (BIRC2) inhibits apoptosis by binding to tumour necrosis factor receptor-associated factors [19] and has been identified as a key driver of aberrant proliferation, multidrug resistance and immune escape of tumour cells [20–23]. BIRC2-mediated evasion of apoptosis plays a key role in the development of HCC [24–26]. Dysregulation of ubiquitination has been associated with aberrant degradation of BIRC2 and apoptotic escape of cancer cells [10, 27, 28]. Although some studies have confirmed that the stability of the BIRC2 protein is regulated by the ubiquitin–proteasome system, studies on the ubiquitination of BIRC2 are limited. Therefore, investigating the role of BIRC2 stabilisation in HCC progression is important for understanding the mechanisms underlying the apoptotic escape of HCC cells.

UBR4, a member of the UBR box E3 ubiquitin protein ligase family, is necessary for the post-translational regulation of eukaryotic proteins [29]. Previous studies have reported that UBR4 is associated with proteasomal degradation, apoptosis, autophagy, yolk sac development and muscle hypertrophy [30–32]. However, the molecular and cellular functions of UBR4 remain elusive because, unlike other E3 ubiquitin ligases, UBR4 does not possess a ring-finger structure or an HECT structural domain but binds to destabilising N-terminal residues (N-degrees) that mediate the degradation of substrate proteins through ubiquitination [30]. Recent studies have reported that UBR4 is associated with the regulation of the circadian rhythm and apoptosis [33, 34]. However, the role of UBR4 in the progression of HCC remains elusive. Although NAP1L1, BIRC2 and UBR4 have been associated with apoptosis, the relationship among the three factors remains unclear. In our previous study, we found a correlation between NAP1L1 and UBR4 and between UBR4 and BIRC2. Therefore, we hypothesised that NAP1L1 affects the stability of the BIRC2 protein through UBR4, which in turn regulates apoptosis and determines the progression of HCC.

We have previously validated that abnormally high expression of NAP1L1 enhances the expression of CCND1 and promotes the proliferation of HCC cells by recruiting HDGF to regulate the function of c-Jun [13]. In this study, we found that NAP1L1 did not affect the migratory and invasive abilities of HCC cells but influenced the stability of the BIRC2 protein through the E3 ubiquitin ligase UBR4, regulating the occurrence of apoptosis and determining the progression of HCC. Altogether, this study reveals a novel mechanism through which NAP1L1 influences the ubiquitination of BIRC2 through UBR4 and regulates apoptosis to determine the progression of HCC. This mechanism represents a promising strategy for targeting NAP1L1 to treat HCC characterised by high expression of NAP1L1 or BIRC2.

## Results

### NAP1L1 promotes HCC cell proliferation and inhibits apoptosis but does not affect the invasive and migratory abilities of HCC cells

**Fig. 1:**
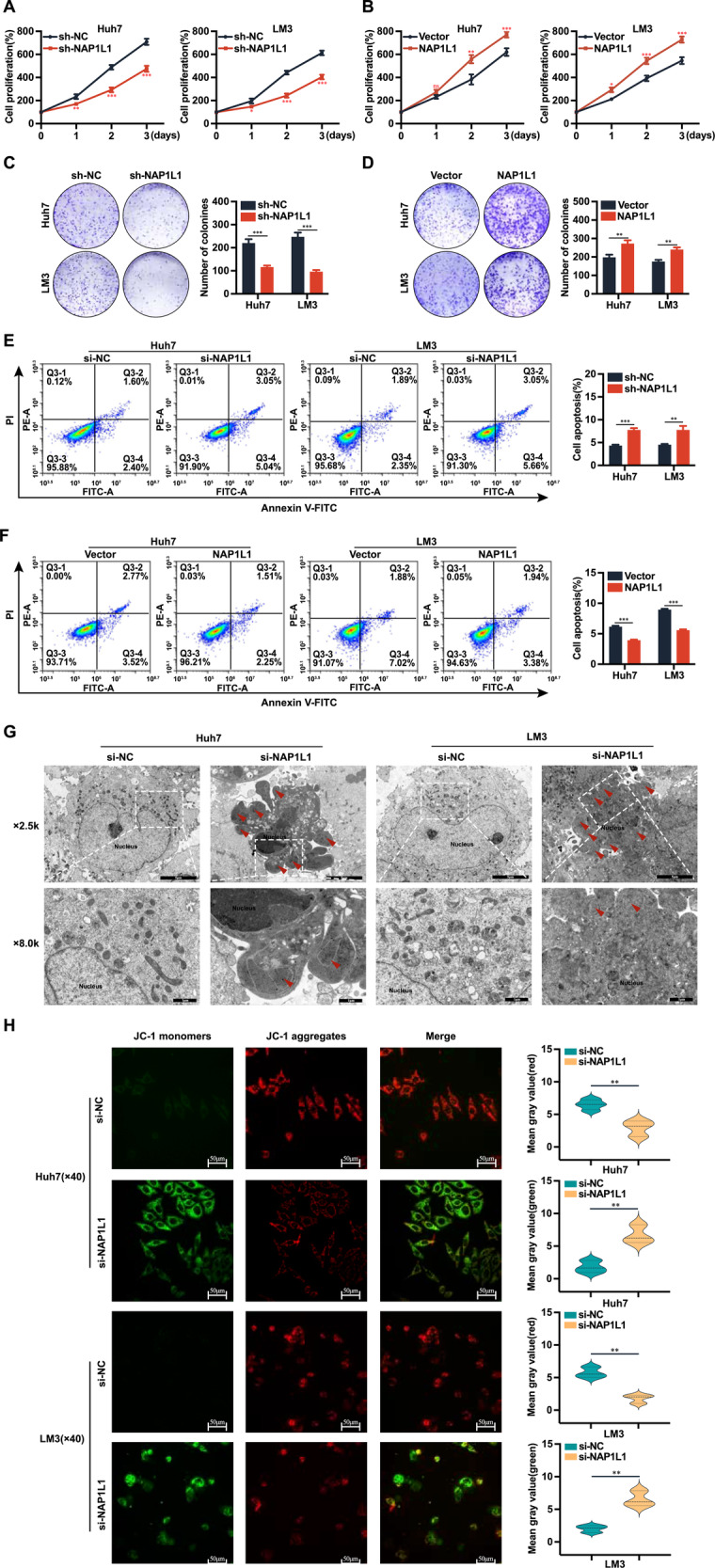
NAP1L1 promotes HCC cell proliferation and inhibits apoptosis. **A**, **B** CCK-8 assay was used to assess the proliferation of Huh7 and LM3 cells after knockdown or overexpression of NAP1L1. **C**, **D** Colony formation assay was performed to assess the ability of Huh7 and LM3 cells to form clones after knockdown or overexpression of NAP1L1. **E**, **F** Flow cytometry was used to assess the apoptosis levels of Huh7 and LM3 cells after knockdown or overexpression of NAP1L1. **G** Transmission electron microscopy was used to observe the morphological and apoptotic features of Huh7 and LM3 cells after knockdown of NAP1L1 (red arrows indicate apoptotic bodies; ×2.5k visual field, scale bar = 5 μm; ×8.0k, scale bar = 1 μm). **H** Laser confocal microscopy was used to assess the mitochondrial membrane potential of Huh7 and LM3 cells after knockdown of NAP1L1. Red fluorescence indicates normal mitochondrial membrane potential, whereas green fluorescence indicates depolarised mitochondrial membrane potential (×40 visual field, scale bar = 50 μm). Data are representative of three independent experiments and are expressed as the mean ± SD (*p < 0.05 versus control; **p < 0.01; ***p < 0.001).

To investigate the effects of NAP1L1 on the malignant behaviour of HCC cells, lentiviral vectors encoding shRNA-NAP1L1 or NAP1L1-overexpression plasmids were transfected into Huh7 and LM3 cells. The transfection efficiency was verified via qRT-PCR at the mRNA level (Supplementary Fig. 1A–D) and western blotting at the protein level (Supplementary Fig. 1E–H). The results of CCK-8 assay (Fig. 1A, B) and colony formation assay (Fig. 1C, D) showed that knockdown of NAP1L1 inhibited the proliferation of HCC cells, whereas overexpression of NAP1L1 had the opposite effect. These results were consistent with those of our previous study [13].

Flow cytometry revealed that knockdown of NAP1L1 significantly increased the apoptosis levels of HCC cells (Fig. 1E), whereas overexpression of NAP1L1 had the opposite effect (Fig. 1F). Similarly, transmission electron microscopy revealed that knockdown of NAP1L1 resulted in an increased number of apoptotic vesicles, a decreased cell volume, nuclear chromatin condensation and occasional vacuole formation in the cytoplasm, indicating an increase in apoptosis levels (Fig. 1G). Laser confocal microscopy revealed that the mitochondrial membrane potential of wild-type HCC cells was normal (red fluorescence), whereas that of NAP1L1-knockdown cells was depolarised (green fluorescence) (Fig. 1H).

Transwell migration and wound healing assays validated that knockdown or overexpression of NAP1L1 had no significant effects on the invasive and migratory abilities of HCC cells (Supplementary Fig. 1 I–L).

### NAP1L1 affects HCC cell apoptosis through the caspase pathway and BIRC2 may be a downstream target of NAP1L1

**Fig. 2:**
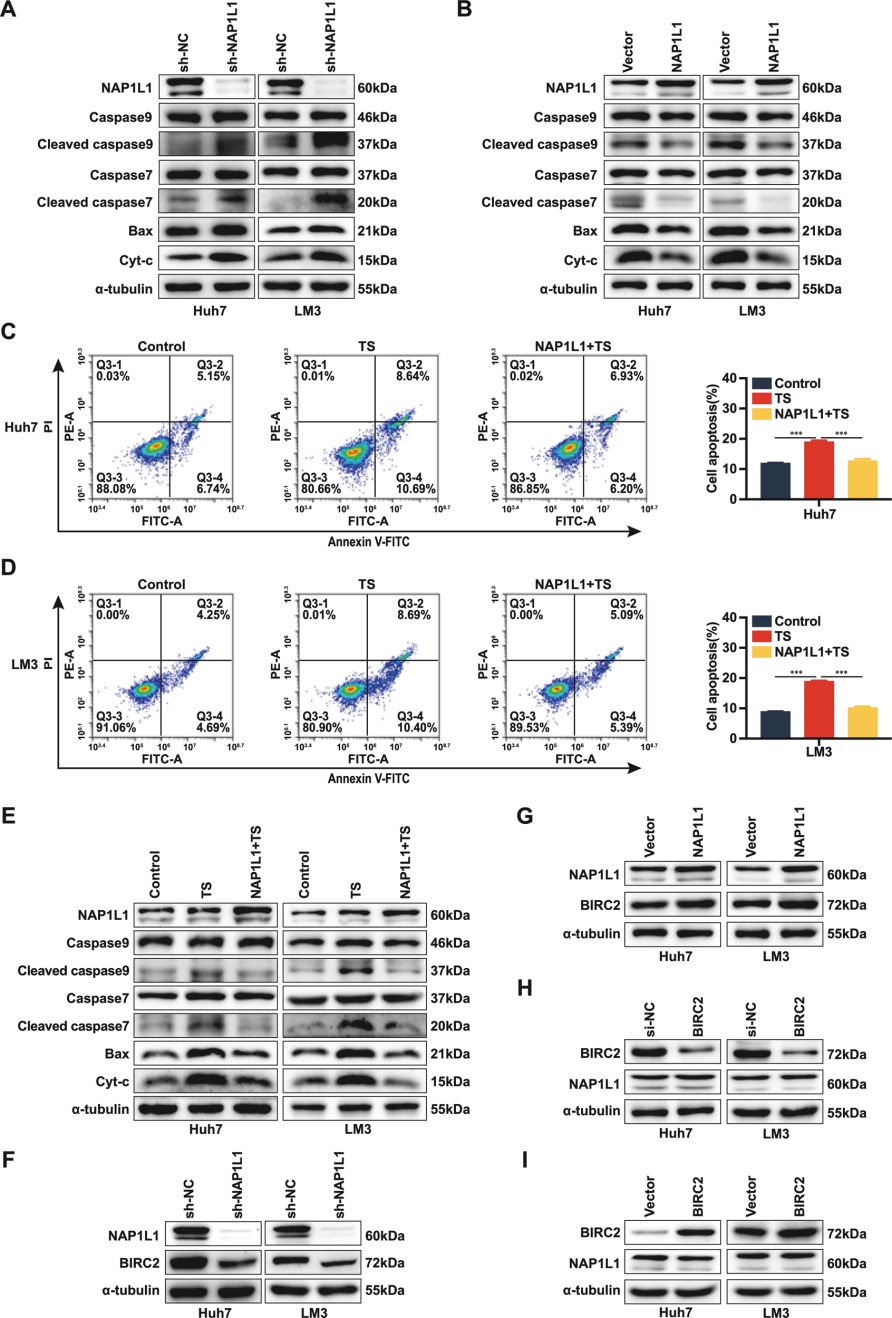
NAP1L1 affects hepatocellular carcinoma cell apoptosis through the caspase pathway and BIRC2 is a downstream target of NAP1L1. **A**, **B** Western blotting was performed to evaluate the expression of apoptosis-related proteins in Huh7 and LM3 cells after knockdown or overexpression of NAP1L1. **C**, **D** Flow cytometry was performed to evaluate apoptosis levels in Huh7 and LM3 cells treated with the apoptosis inducer TS and/or NAP1L1-overexpression plasmids. **E** Western blotting was performed to evaluate the expression of apoptosis-related proteins in Huh7 and LM3 cells treated with the apoptosis inducer TS and/or NAP1L1-overexpression plasmids. **F**–**I** Western blotting assay was performed to evaluate the protein expression of NAP1L1 and BIRC2 after knockdown or overexpression of NAP1L1/BIRC2. Data are representative of three independent experiments and are expressed as the mean ± SD (*p < 0.05 versus control; **p < 0.01; ***p < 0.001).

The expression of the apoptotic proteins Bax, Cyt-c, cleaved caspase 9 and cleaved caspase 7 was significantly upregulated after knockdown of NAP1L1 (Fig. 2: A) and downregulated after overexpression of NAP1L1 (Fig. 2B), suggesting that NAP1L1 affects cell apoptosis via a caspase-dependent pathway. To verify this hypothesis, we treated HCC cells with an apoptosis inhibitor (Z-VAD-FMK, ZVF) or an apoptosis inducer (TNF-α + SM-164, TS). Flow cytometry showed that overexpression of NAP1L1 effectively inhibited TS-induced apoptosis (Fig. 2C, D). Similarly, ZVF inhibited the NAP1L1 knockdown-induced apoptosis of HCC cells (Supplementary Fig. 2A, B). WB showed that TS significantly increased the expression of Bax, Cyt-c, cleaved caspase 9 and cleaved caspase 7, whereas overexpression of NAP1L1 counteracted the effects of TS (Fig. 2E). Similarly, knockdown of NAP1L1 significantly increased the expression of Bax, Cyt-c, cleaved caspase 9 and cleaved caspase 7, whereas ZVF reversed the effects of NAP1L1 knockdown (Supplementary Fig. 2C). These results suggest that NAP1L1 affects the apoptosis of HCC cells through a caspase-dependent pathway.

BIRC2 plays a crucial role as an apoptosis inhibitor during tumour cell development. Recent studies have shown that BIRC2 is responsible for the apoptotic escape and multidrug resistance of tumour cells [20]. In this study, the protein expression of BIRC2 was positively correlated with that of NAP1L1. In particular, knockdown of NAP1L1 decreased the protein expression of BIRC2 (Fig. 2F), whereas overexpression of NAP1L1 had the opposite effect (Fig. 2G). However, overexpression or knockdown of NAP1L1 had no significant effects on the mRNA expression of BIRC2 (Supplementary Fig. 2D~E). Moreover, overexpression or transient knockdown of BIRC2 had no significant effects on the protein expression of NAP1L1 (Fig. 2H, I). These results suggest that BIRC2 is a downstream target of NAP1L1 and that NAP1L1 regulates the protein expression of BIRC2 through post-translational modifications (e.g. ubiquitination).

To explore the relationship between NAP1L1 and BIRC2 proteins and apoptosis, we first identified the differential genes associated with NAP1L1 and BIRC2 in hepatocellular carcinomas in the TCGA database using single-gene-differential-analysis, and then functionally clustered these differential genes using GO/KEGG combined with logFC. It was found that both NAP1L1 and BIRC2 were involved in the negative regulation of the execution phase of apoptosis (Table 1), and details of the other clusters are shown in Additional file 1 and Additional file 2.

**Table 1:** GO/KEGG combined logFC functional clustering analysis of single-gene related differential genes.

Gene name	ID	Description	*P* value
NAP1L1	GO:1900117	Regulation of execution phase of apoptosis	0.0032
	GO:1900118	Negative regulation of execution phase of apoptosis	0.0002
BIRC2	GO:1900117	Regulation of execution phase of apoptosis	0.0010
	GO:1900118	Negative regulation of execution phase of apoptosis	0.0002

### BIRC2 is upregulated in HCC tissues and is associated with a poor prognosis

Analysis of TCGA data showed that the mRNA expression of BIRC2 was significantly higher in HCC tissues than in normal liver tissues. The high expression of BIRC2 was significantly associated with a poor prognosis in patients with HCC (Supplementary Fig. 3A, B). Consistently, immunohistochemical analysis revealed that BIRC2 expression was significantly higher in HCC tissues than in adjacent normal tissues (Supplementary Fig. 3C, D).

**Fig. 3:**
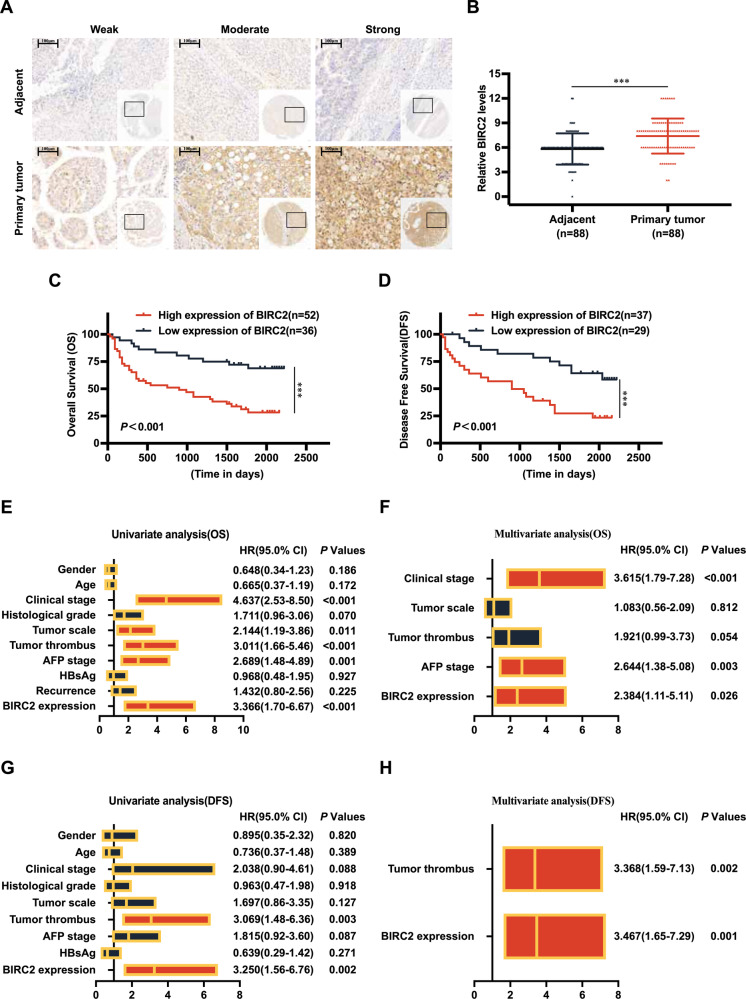
BIRC2 is upregulated in HCC tissues and is associated with a poor prognosis. **A** TMA-based analysis of BIRC2 expression (magnification, ×10 and ×40; scale bar = 100 μm). **B** Immunohistochemical staining scores of TMA. **C**, **D** Kaplan–Meier analysis of overall survival (OS) and disease-free survival (DFS) in TMA with high BIRC2 expression. **E**, **F** Summary of univariate and multivariate Cox regression analyses of OS. **G**, **H** Summary of univariate and multivariate Cox regression analyses of DFS. Data are representative of three independent experiments and are expressed as the mean ± SD (*p < 0.05 versus control; **p < 0.01; ***p < 0.001).

Furthermore, immunohistochemical staining was performed on 90 pairs of HCC and adjacent non-tumour tissue samples using TMA. Two pairs of tissue samples underwent desludging during the staining process and were excluded from further analysis. Eventually, a total of 88 pairs of tissue samples were included for immunohistochemical scoring. The results revealed that BIRC2 expression was significantly higher in HCC tissues than in adjacent normal tissues (Fig. 3A, B). Survival analysis showed that high BIRC2 expression was a risk factor for prognosis, as it was associated with poor overall survival and disease-free survival (Fig. 3C, D). Furthermore, BIRC2 expression was significantly associated with clinical characteristics such as clinical stage, tumour size and survival status in HCC (Table 2). Univariate and multivariate Cox regression analyses showed that BIRC2 was an independent risk factor for overall survival and disease-free survival in HCC (Fig. 3E–H). These results suggest that high expression of BIRC2 is closely associated with tumour progression in HCC.

**Table 2:** Correlation of BIRC2 expression with clinicopathological characteristics of HCC.

Characteristics	n	BIRC2 expression		
		High	Low	*P*
Gender				
Male	68	37 (54.41%)	31 (45.59%)	0.100
Female	20	15 (75.00%)	5 (25.00%)	
Age (year)				
≤50	36	22 (61.11)	14 (38.89%)	0.748
>50	52	30 (57.69%)	22 (42.31%)	
Clinical stage				
I~II	69	34 (49.28%)	35 (50.72%)	<0.001
III~IV	19	18 (94.74%)	1 (5.26%)	
Histological grade				
I~II	51	26 (50.98%)	25 (49.02%)	0.069
III	37	26 (70.27%)	11 (29.73%)	
Tumor scale(cm)				
≤5	49	22 (44.90%)	27 (55.10%)	0.002
>5	39	30 (76.92%)	9 (23.08%)	
Tumor thrombus				
No	61	33 (54.10%)	28 (45.90%)	0.152
Yes	27	19 (70.37%)	8 (29.63%)	
AFP stage(μg/L)				
≤200	46	25 (54.35%)	21 (45.65%)	0.344
>200	42	27 (64.29%)	15 (35.71%)	
HBsAg				
Negative	18	10 (55.56%)	8 (44.44%)	0.732
Positive	70	42 (60.00%)	28 (40.00%)	
Recurrence				
No	54	29 (53.70%)	25 (46.30%)	0.195
Yes	34	23 (67.65%)	11 (32.35%)	
Vital states				
Alive	42	17 (40.48%)	25 (59.52%)	0.001
Die	46	35 (76.09)	11 (23.91%)	

### BIRC2 promotes HCC cell proliferation and inhibits apoptosis

**Fig. 4:**
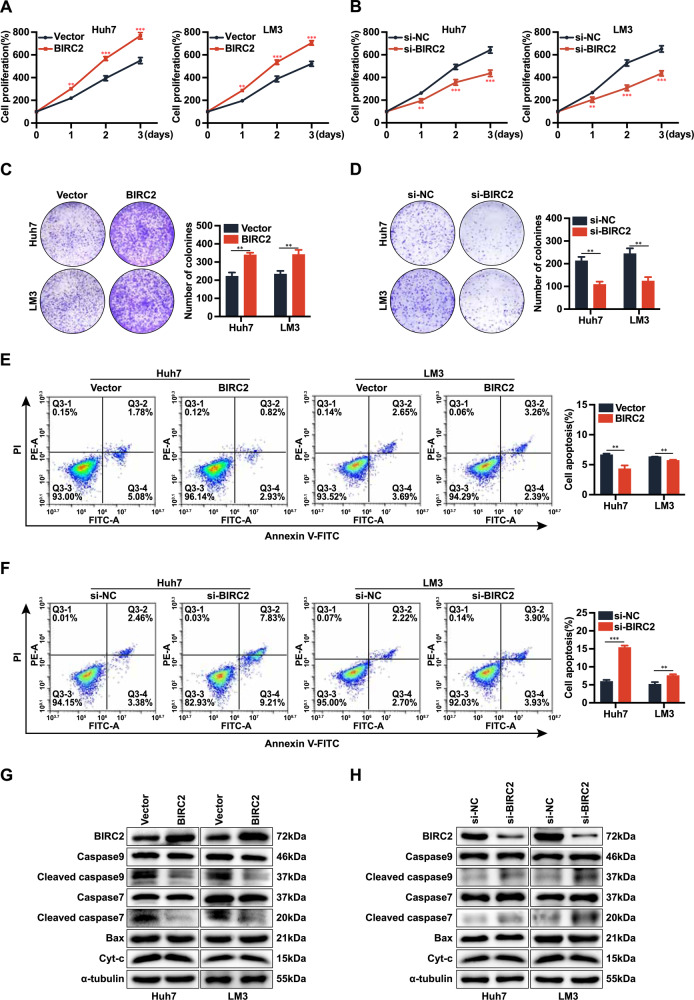
BIRC2 promotes HCC cell proliferation and inhibits apoptosis. **A**, **B** CCK-8 assay was performed to assess the proliferation of Huh7 and LM3 cells after overexpression or knockdown of BIRC2. **C**, **D** Colony formation assay was performed to assess the ability of Huh7 and LM3 cells to form clones after overexpression or knockdown of BIRC2. **E**, **F** Flow cytometry was performed to evaluate the apoptosis levels of Huh7 and LM3 cells after overexpression or knockdown of BIRC2. **G**, **H** Western blotting was performed to evaluate the expression of apoptosis-related proteins in Huh7 and LM3 cells after overexpression or knockdown of BIRC2. Data are representative of three independent experiments and are expressed as the mean ± SD (*p < 0.05 versus control; **p < 0.01; ***p < 0.001).

To examine the effects of BIRC2 on the proliferation and apoptosis of HCC cells, we transfected the cells with BIRC2-overexpression plasmids and siRNAs targeting BIRC2 and verified the transfection efficiency via qRT-PCR and WB (Supplementary Fig. 4A–H). The results of CCK-8 assay (Fig. 4A, B) and colony formation assay (Fig. 4C, D) showed that overexpression of BIRC2 significantly enhanced the proliferative ability of HCC cells, whereas transient knockdown of BIRC2 had the opposite effect. Flow cytometry revealed that overexpression of BIRC2 significantly reduced the apoptosis levels of HCC cells, whereas knockdown of BIRC2 had the opposite effect (Fig. 4E–F). In addition, western blotting showed that BIRC2 inhibited apoptosis by reducing the expression of cleaved caspase 9 and cleaved caspase 7, but had no effect on the expression of Bax and Cyt-c proteins (Fig. 4G, H).

### Knockdown of NAP1L1 upregulates UBR4 expression to promote ubiquitin-mediated degradation of BIRC2

**Fig. 5:**
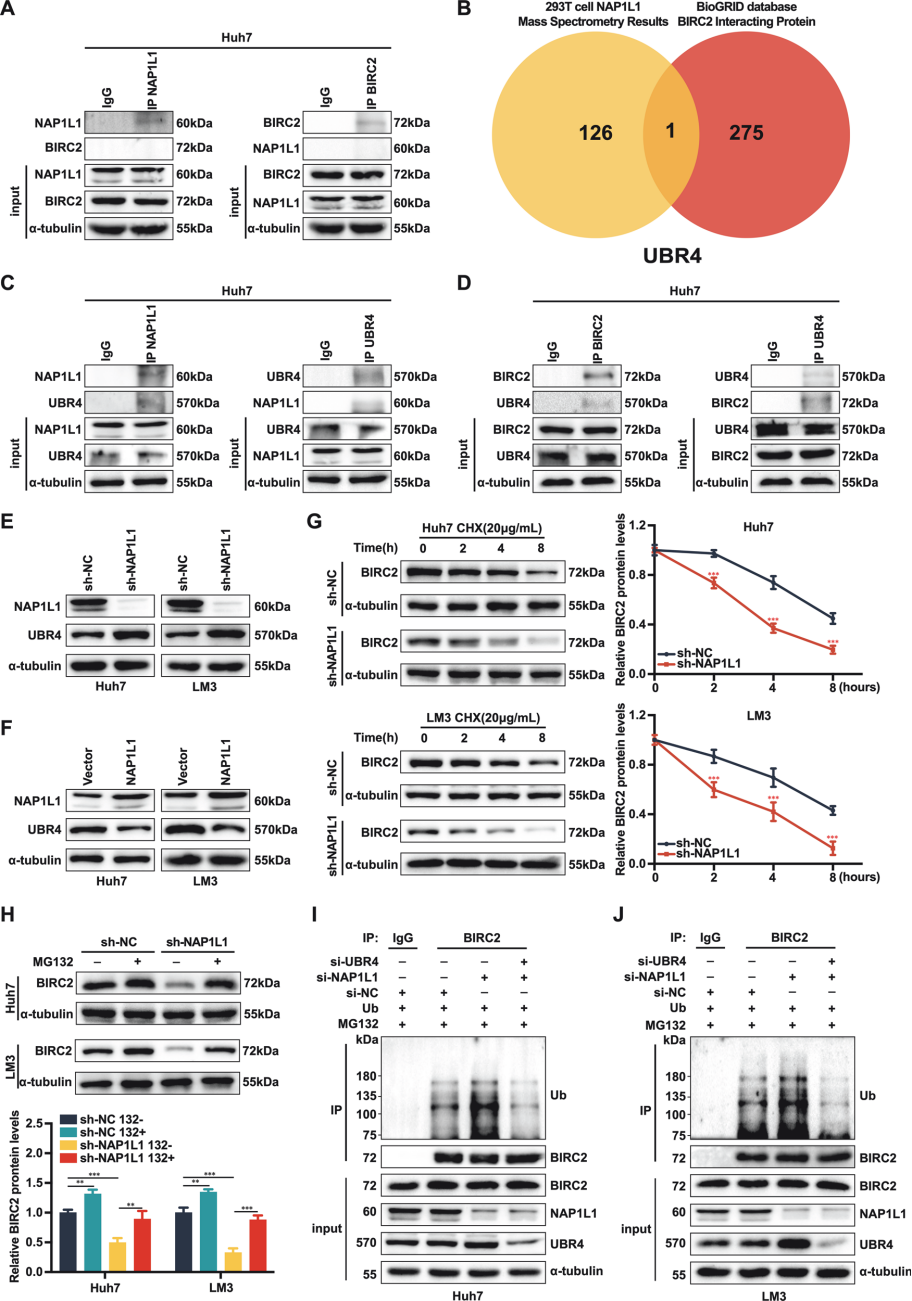
Knockdown of NAP1L1 upregulates UBR4 expression, which in turn promotes the ubiquitin-mediated degradation of BIRC2. **A** Co-IP assays revealed no direct binding between NAP1L1 and BIRC2 proteins. **B** Venn diagram demonstrating the intersection of overexpressed NAP1L1 (mass spectrometry data) and BIRC2-associated proteins. **C** Co-IP assay revealed that NAP1L1 could bind to UBR4. **D** Co-IP assay revealed that BIRC2 could bind to UBR4. **E**, **F** Western blotting was performed to evaluate the protein expression of NAP1L1 and UBR4 in Huh7 and LM3 cells after knockdown or overexpression of NAP1L1. **G** Western blotting was performed to evaluate the protein expression of BIRC2 in Huh7/LM3 cells transfected with sh-NAP1L1 and treated with cycloheximide (CHX, 0.2 mg/mL) at indicated time points. Signals for BIRC2 were quantified via densitometric analysis, and the relative abundance of BIRC2 protein at the time of CHX addition (0 h) was set to 1. **H** Protein expression of BIRC2 in Huh7/LM3 cells transfected with shNAP1L1 and treated with MG132 as indicated. **I**, **J** BIRC2 ubiquitination in NAP1L1-knockdown Huh7 and LM3 cells co-transfected with siUBR4 and HA-Ub plasmids. The transfected cells were treated with MG132 (20 μM for 6 h) before being harvested. Data are representative of three independent experiments and are expressed as the mean ± SD (*p < 0.05 versus control; **p < 0.01; ***p < 0.001).

To examine whether NAP1L1 binds to BIRC2, mass spectrometry was performed on 293T cells transfected with NAP1L1-overexpression plasmids. A total of 127 proteins were found to bind to NAP1L1. However, these proteins did not include BIRC2 (Additional file 3). The results of Co-IP were consistent with those of mass spectrometry (Fig. 5A), suggesting that NAP1L1 regulates the protein expression of BIRC2 through intermediate molecules. The BioGRID database was used to predict intermediate proteins associating NAP1L1 with BIRC2. Based on the prediction results and mass spectrometry data, a Venn diagram was generated, which demonstrated that UBR4 was an intermediate molecule associating NAP1L1 with BIRC2 (Fig. 5B). The results of Co-IP validated the binding of NAP1L1 to UBR4 and that of UBR4 to BIRC2 (Fig. 5C, D). In addition, the protein expression of UBR4 was negatively correlated with that of NAP1L1. Knockdown of NAP1L1 increased the protein expression of UBR4, whereas overexpression of NAP1L1 had the opposite effect (Fig. 5E, F).

To elucidate the specific mechanism through which NAP1L1 affects BIRC2, HCC cells were treated with actinomycin ketone (CHX), proteasome inhibitor MG132, lysosomal inhibitors chloroquine and 3-methyladenine (3-MA). The BIRC2 protein was destabilised and the rate of protein degradation was significantly increased in cells with NAP1L1 knockdown and CHX treatment (Fig. 5G). Treatment with MG132 significantly inhibited the degradation of BIRC2 protein (Fig. 5H), However, chloroquine and 3-MA did not have similar effects (Supplementary Fig. 4I, J). These results suggest that NAP1L1 affects the protein expression of BIRC2 through the ubiquitin–proteasome system. Furthermore, knockdown of NAP1L1 significantly enhanced the ubiquitination of the BIRC2 protein, whereas the simultaneous knockdown of UBR4 and NAP1L1 had the opposite effect (Fig. 5I, J). These results indicated that knockdown of NAP1L1 upregulated the expression of the E3 ligase UBR4, which in turn promoted the ubiquitin-mediated degradation of BIRC2.

### Overexpression of BIRC2 promotes HCC cell proliferation and reverses apoptosis induced by knockdown of NAP1L1

**Fig. 6:**
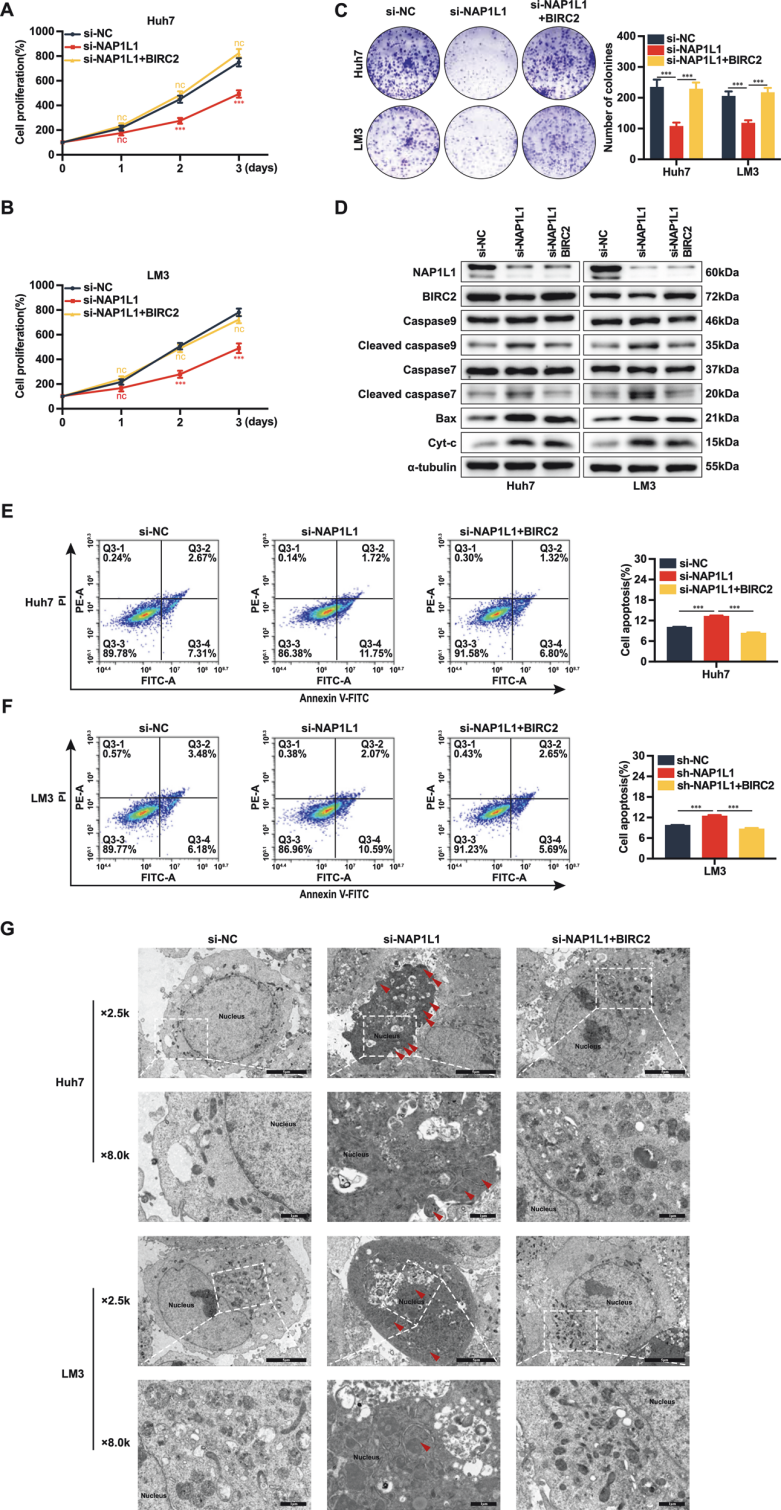
Overexpression of BIRC2 promotes HCC cell proliferation and reverses apoptosis induced by knockdown of NAP1L1. **A**, **B** CCK-8 assay was performed to assess the effects of BIRC2 overexpression on the proliferation of Huh7 and LM3 cells with NAP1L1 knockdown. **C** Colony formation assay was performed to assess the effects of BIRC2 overexpression on the colony-forming ability of Huh7 and LM3 cells with NAP1L1 knockdown. **D** Western blotting was performed to assess the effects of BIRC2 overexpression on the expression of apoptotic proteins in Huh7/LM3 cells with NAP1L1 knockdown. **E**, **F** Flow cytometry was performed to assess the effects of BIRC2 overexpression on the apoptosis levels of Huh7/LM3 cells with NAP1L1 knockdown. **G** Transmission electron microscopy was performed to assess the effects of BIRC2 overexpression on the morphological and apoptotic features of Huh7/LM3 cells with NAP1L1 knockdown. Data are representative of three independent experiments and are expressed as the mean ± SD (*p < 0.05 versus control; **p < 0.01; ***p < 0.001).

To investigate whether NAP1L1 affects apoptosis through BIRC2, we transfected plasmids overexpressing BIRC2 based on sh-NAP1L1. CCK-8 assay (Fig. 6A, B) and colony formation assay (Fig. 6C) revealed that the proliferative ability of knockdown HCC cells was restored after overexpression of BIRC2. Western blotting revealed that the protein expression of Bax, Cyt-c, cleaved caspase 9 and cleaved caspase 7 was significantly higher in the sh-NAP1L1 group than in the sh-NC group, whereas that of cleaved caspase 9 and cleaved caspase 7 was significantly lower in the sh-NAP1L1 + BIRC2-overexpression group than in the sh-NAP1L1 group. However, no significant changes were observed in the protein expression of Bax and Cyt-c (Fig. 6D).

**Fig. 7:**
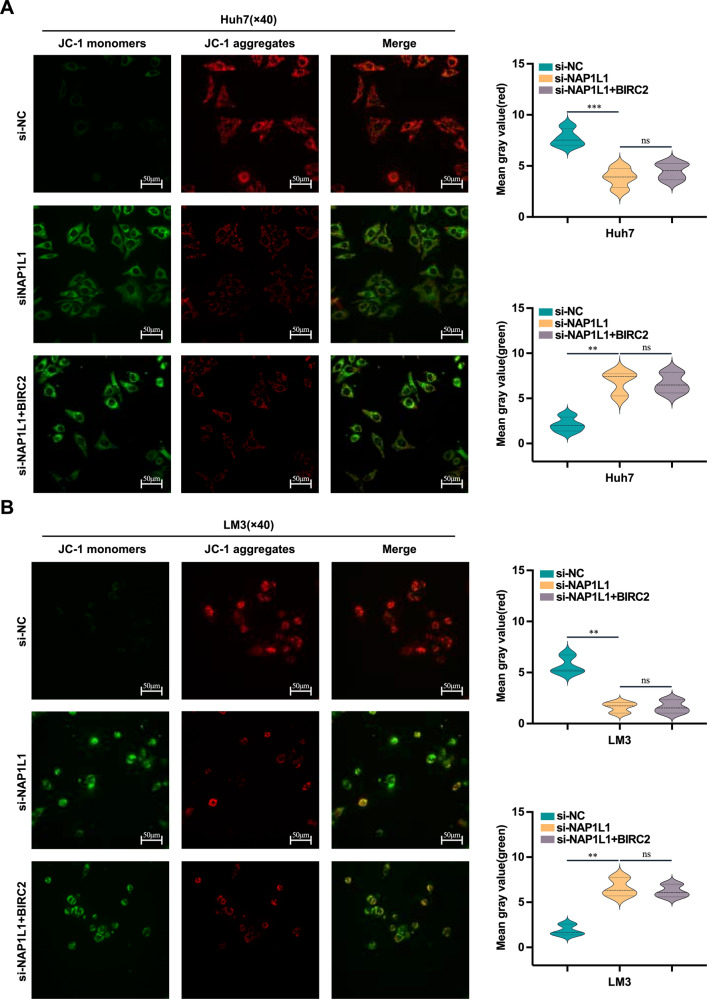
Overexpression of BIRC2 reverses the NAP1L1 knockdown-induced decrease in mitochondrial membrane potential. **A**, **B** Laser confocal microscopy was performed to assess the effects of BIRC2 overexpression on the mitochondrial membrane potential of Huh7/LM3 cells with NAP1L1 knockdown. Red fluorescence indicates normal mitochondrial membrane potential, whereas green fluorescence indicates depolarised mitochondrial membrane potential (×40 visual field, scale bar = 50 μm). Data are representative of three independent experiments and are expressed as the mean ± SD. (*p < 0.05 versus control; **p < 0.01; ***p < 0.001).

Flow cytometry showed that knockdown of NAP1L1 significantly enhanced the apoptosis of HCC cells, whereas overexpression of BIRC2 counteracted this effect (Fig. 6E, F). The results of transmission electron microscopy were consistent with those of flow cytometry (Fig. 6G). Depolarisation of mitochondrial membrane potential is a hallmark of caspase-dependent apoptosis [35]. However, laser confocal microscopy revealed that overexpression of BIRC2 did not affect the NAP1L1 knockdown-induced depolarisation of mitochondrial membrane potential in HCC cells (Fig. 7A, B).

### Knockdown of NAP1L1 inhibits tumour growth and promotes the protein expression of cleaved caspase 9

**Fig. 8:**
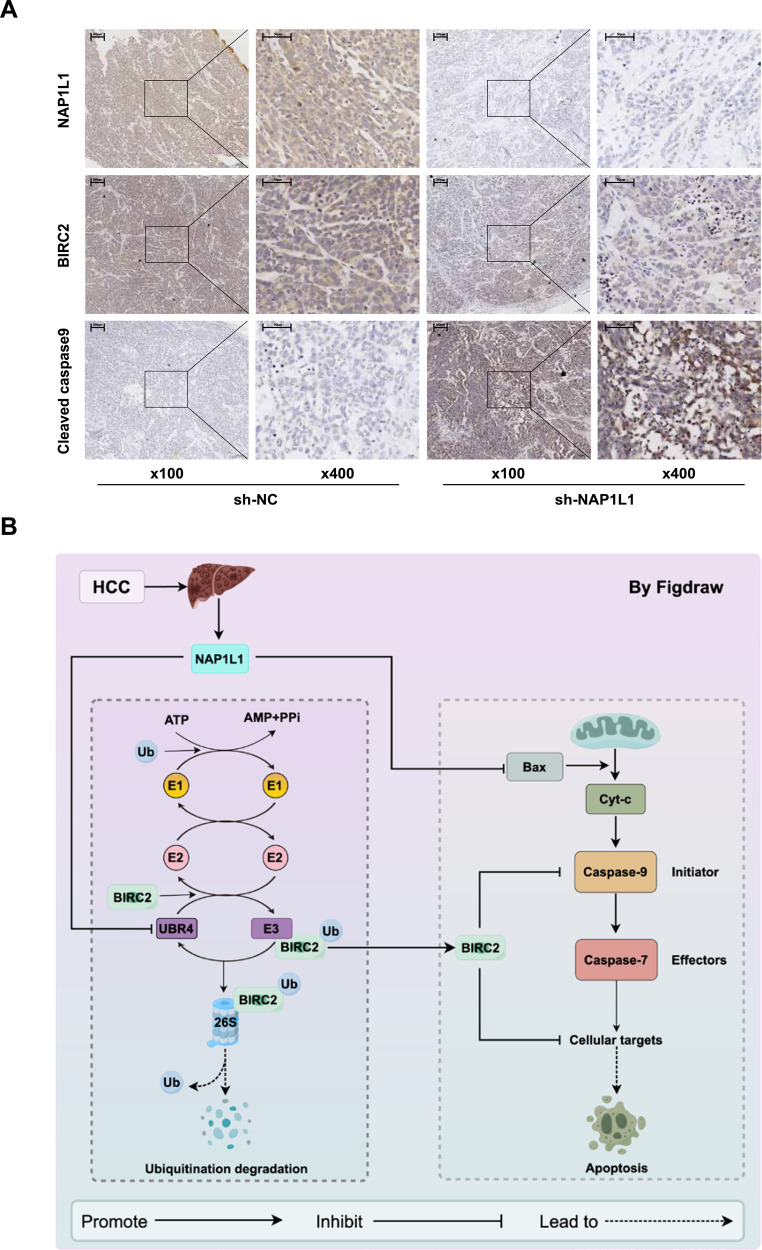
Knockdown of NAP1L1 inhibits tumour growth and promotes the expression of the apoptotic protein cleaved caspase 9 in HCC. **A** Immunohistochemical staining was performed to detect the expression of NAP1L1, BIRC2 and cleaved caspase 9 in xenograft tumour tissues from nude mice (×100 visual field, scale bar = 100 μm; ×400, scale bar = 50 μm). **B** NAP1L1 is highly expressed in HCC cells and tissues, and upregulated NAP1L1 directly inhibits the expression of apoptotic proteins, such as Bax and Cyt-c, thereby inducing the apoptotic escape of HCC cells. In addition, NAP1L1 plays a pro-tumorigenic role by inhibiting the expression of the E3 ubiquitin ligase UBR4, thereby reducing the ubiquitin-mediated degradation of BIRC2 and inhibiting the activation of the caspase pathway.

In a previous study, we found that knockdown of NAP1L1 significantly reduced tumorigenicity and the protein expression of PCNA and Ki67 in mice in vivo [13]. In this study, we performed immunohistochemical analysis using tumour tissues from mice in the sh-NC and sh-NAP1L1 groups in the aforementioned study. The results revealed that the expression of NAP1L1 and BIRC2 was significantly lower and that of cleaved caspase 9 was significantly higher in the sh-NAP1L1 group than in the sh-NC group (Fig. 8A).

## Discussion

With the continuous development of modern medicine, remarkable progress has been made in cancer treatment; however, the prognosis of HCC remains poor [36, 37]. The median survival of patients with HCC is only 24 months, and the 5-year survival rate is only 16%. This poor survival is mainly attributed to the apoptotic escape and multidrug resistance of tumour cells [38, 39]. However, the mechanisms underlying the apoptotic escape of HCC cells remain unclear. Elucidating these mechanisms may help to understand the progression of HCC. NAP1L1 has been shown to play a key role in the progression of multiple tumours [40–42]. Although NAP1L1 can induce the apoptotic escape of tumour cells [17], whether it determines the progression of HCC by affecting the stability of BIRC2, a key factor in apoptotic escape, remains unclear.

We have previously shown that NAP1L1 plays a crucial role in the progression of human HCC and its upregulated expression is associated with a poor prognosis in patients with HCC. Upregulated NAP1L1 plays a pro-tumorigenic role by recruiting HDGF to interact with c-Jun, thereby upregulating the expression of CCND1 and promoting the proliferation of tumour cells [13]. In this study, both in vivo and in vitro experiments demonstrated that knockdown of NAP1L1 significantly inhibited HCC cell proliferation and promoted HCC cell apoptosis. These results suggest that NAP1L1 plays an oncogenic role in the progression of HCC, which is consistent with the results of previous studies [14, 15, 40]. On examining the effects of NAP1L1 on other malignant behaviours of HCC cells, we found that NAP1L1 had no significant effects on the migratory and invasive abilities of HCC cells.

Knockdown of NAP1L1 significantly downregulated the protein expression of BIRC2 (an anti-apoptotic factor) but did not affect its mRNA expression in Huh7 and LM3 cells. These results suggest that NAP1L1 affects BIRC2 activity through post-translational regulation (e.g. degradation by the ubiquitin–proteasome system) instead of translational regulation. Previous studies have reported that NAP1L1 does not affect ubiquitination but can inhibit the ubiquitin-mediated degradation of Yes-associated protein 1 (YAP1) and regulate cardiomyocyte fibrosis [18]. In this study, the results of Co-IP validated that NAP1L1 did not bind to BIRC2, suggesting the involvement of intermediate molecules in the regulatory effects of NAP1L1 on BIRC2. A recent study showed that UBR4, an E3 ubiquitin ligase, is involved in the apoptosis of renal cancer cells [43]. Bioinformatic analysis revealed that BIRC2 could bind to UBR4, whereas mass spectrometry revealed that NAP1L1 could bind to UBR4 in HCC cells with overexpression of NAP1L1. Therefore, we hypothesised that NAP1L1 regulated the stability of the BIRC2 protein by affecting the expression of UBR4, which is involved in the activation or inhibition of apoptosis and hence determines the progression of HCC. Knockdown of NAP1L1 significantly increased the protein expression of UBR4 and the ubiquitination level of BIRC2. Transient knockdown of UBR4 in HCC cells with knockdown of NAP1L1 significantly reduced the ubiquitination level of BIRC2, suggesting that NAP1L1 regulates the ubiquitination of BIRC2 through UBR4.

BIRC2, a member of the IAP family of apoptosis inhibitory proteins, was initially identified in the tumour necrosis factor receptor 2 (TNFR2) complex and was subsequently detected in all human tissues [44, 45]. During TNFR-mediated activation of nuclear factor-kappa B (NF-κB), the increased expression of BIRC2 effectively reduces caspase 8 activity, which in turn protects against TNF-induced apoptosis [46, 47]. BIRC2 has been identified as an E3 ubiquitin ligase in the ubiquitin–proteasome system that mediates the ubiquitin-mediated degradation of caspase 8, caspase 7 and caspase 3 and exerts an anti-apoptotic effect [48–50]. Semenza et al. [20] found that overexpression of BIRC2 in tumour tissues was a key marker of immunotherapy resistance and that silencing of BIRC2 significantly reduced the tumorigenicity of breast cancer and melanoma cells. BIRC2 is highly expressed in various tumours, including breast cancer, melanoma, glioblastoma, gallbladder cancer and nasopharyngeal carcinoma, and plays a crucial role in the escape of tumour cells from apoptosis [51–54]. In this study, analysis of TCGA data revealed that BIRC2 was highly expressed in HCC tissues and was associated with a poor prognosis. However, the molecular mechanisms through which BIRC2 regulates the apoptosis of HCC cells remain unclear. Immunohistochemical staining using TMA revealed that the expression of BIRC2 was significantly higher in HCC tissues than in adjacent paracancerous tissues. In addition, the high expression of BIRC2 was associated with a poor prognosis in patients with HCC. Univariate and multivariate Cox analyses demonstrated that BIRC2 was an independent risk factor affecting overall survival and disease-free survival in HCC. These results are consistent with those obtained from analysis of TCGA data. Knockdown of BIRC2 inhibited the proliferative ability of HCC cells and activated the apoptosis of HCC cells. These results suggest that BIRC2 plays a pro-carcinogenic role in HCC, which is consistent with its role in other tumours [55, 56]. Interestingly, our experimental results revealed that altered BIRC2 expression could affect the expression of the downstream proteins Cleaved caspase9 and Cleaved caspase7, but had no significant effect on the expression of Bax and Cyt-c proteins. Bax protein is a pro-apoptotic protein located on the outer mitochondrial membrane, whose activation or aggregation can lead to mitochondrial permeable membrane changes, which can regulate Cyt C release from mitochondria into cytosol, thereby inducing induces apoptosis [57]. Bax is mainly regulated by the anti-apoptotic protein Bcl-2. Under normal conditions, the Bax protein is in an inactive state, while the Bcl-2 protein maintains the survival state of cells by binding to Bax and inhibiting its activity. When apoptotic signals occurs in cells, Bcl-2 releases Bax, and activated Bax proteins accumulate on the mitochondrial surface to form pores, leading to changes in mitochondrial membrane permeability and ultimately triggering apoptosis [57, 58]. Hence, the pathway by which NAP1L1 regulates apoptosis in HCC cells may not be limited to the BIRC2 signaling pathway, but may also be related to the aberrant expression of the anti-apoptotic protein Bcl-2.

Furthermore, this study showed that NAP1L1 regulated the ubiquitin-mediated degradation of BIRC2 through UBR4, which in turn determined the progression of HCC. However, whether NAP1L1 affects HCC cell apoptosis through BIRC2 remains unclear. Consequently, we transfected BIRC2-overexpression plasmids into HCC cells with NAP1L1 knockdown and verified the apoptosis level, cell morphology, mitochondrial membrane potential and expression of apoptotic proteins via flow cytometry, transmission electron microscopy, laser confocal microscopy and western blotting, respectively. Overexpression of BIRC2 reversed NAP1L1 knockdown-induced apoptosis in HCC cells as evidenced by the downregulation of apoptotic proteins such as cleaved caspase 9 and cleaved caspase 7 and relatively normal cell morphology. However, overexpression of BIRC2 did not affect the depolarisation of mitochondrial membrane potential induced by knockdown of NAP1L1, suggesting that NAP1L1 regulates apoptosis by affecting not only the ubiquitin-mediated degradation of BIRC2 but also the mitochondrial membrane potential.

This study reveals that NAP1L1 regulates HCC cell proliferation and apoptosis but does not affect the migratory and invasive abilities of HCC cells. Mechanistically, NAP1L1 regulates the ubiquitination of BIRC2 to activate or inhibit apoptosis through the E3 ubiquitin ligase UBR4, which in turn determines the progression of HCC (Fig. 8B). This mechanism may represent a novel strategy for targeted treatment of HCC. Tumour progression may be inhibited by suppressing NAP1L1 expression in patients with HCC with high protein expression of NAP1L1 and BIRC2. In conclusion, the findings of this study provide a new theoretical basis for the development of drugs targeting NAP1L1 for the treatment of HCC.

### Limitations

- The specific mechanism underlying the NAP1L1 knockdown-induced increase in the protein expression of UBR4 warrants further investigation.
- A total of 20 pairs of clinical HCC and adjacent normal tissues were collected for immunohistochemical analysis. Given that this is a small sample size, the experimental results may be biased.
- Mouse tumour tissues from our previous study were used for immunohistochemical staining, resulting in a relatively weak chain of evidence for in vivo experiments.

## Materials and methods

### Cell culture

HCC cell lines (Huh7 and HCCLM3) were purchased from the cell bank of the Typical Culture Preservation Committee of the Chinese Academy of Sciences. The cells were cultured in high-glucose Dulbecco’s modified Eagle medium (DMEM) supplemented with 10% foetal bovine serum (FBS) and 1% triple antibiotics (penicillin, streptomycin and gentamicin) in Petri dishes and maintained at 37°C in a thermostat incubator with 5% CO_2_.

### Cell transfection

Huh7 and HCCLM3 cells were inoculated in 6-well plates. When the cells reached approximately 50% confluence, the medium was replaced with DMEM and the cells were infected with Lipofectamine 3000, P3000 reagent and lentiviral vectors or plasmids. The medium was replaced with complete DMEM after 8 hours, and overexpression or knockdown efficiency was verified via real-time reverse transcription polymerase chain reaction (qPCR) and western blotting. The sequences of lentiviral vectors, plasmids and siRNAs used in this study are provided in Supplementary Table 1.

### Cell proliferation assay

HCC cells were inoculated in 96-well plates at a density of 3000 cells/well and cultured at 37°C and 5% CO_2_. Cell viability was assessed using a CCK-8 kit. At 0, 24, 48 and 72 h after culture, the cells were incubated with 10 μL of CCK-8 reagent for 2 h. Subsequently, absorbance was measured at 450 nm using a multifunctional enzyme marker. Three wells were included in each group, and the experiment was repeated three times independently.

### Colony formation assay

HCC cells were inoculated in 6-well plates at a density of 800 cells/well (the number of cells was determined according to the minimum population dependence of the cell line). The cells were cultured in a complete medium at 37°C and 5% CO_2_, and the medium was replaced at an interval of 3 days. After 14 days of culture, the cells were washed twice with PBS, fixed with methanol for 10 min and stained with crystal violet solution for 3 min. The residual stain was washed with tap water, and the cells were allowed to air dry at room temperature. Subsequently, cell colonies were counted under a microscope. The experiment was repeated three times independently.

### Wound healing assay

HCC cells were inoculated in 6-well plates. When the cells reached 80% confluence, the tip of a 10-μL pipette was used to create a linear scratch on the cell monolayer. At 0 and 48 h, cell migration was observed under a microscope, the cells were photographed and the cell migration rate was calculated. The experiment was repeated three times independently.

### Transwell migration assay

Cells were resuspended in serum-free DMEM, and the cell density was adjusted to 1 × 10^5^ cells/mL. A total of 500 μL of DMEM supplemented with 10% FBS was added to the lower transwell chamber, whereas 200 μL of the cell suspension was added to the upper chamber. The upper chamber was placed into the lower chamber, and the cells were incubated in a humidified incubator with 5% CO_2_ at 37 °C for 24 h. Subsequently, non-migratory cells in the upper chamber were removed using a cotton swab, whereas migratory cells at the bottom of the chamber were fixed with 4% paraformaldehyde for 20 min and stained with 0.1% crystal violet for 5 min. The number of migratory cells in each field of view was counted under a microscope. The experiment was repeated three times independently.

### Apoptosis assay

Apoptosis was assessed using the Annexin V-FITC/PI Apoptosis Detection Kit. Briefly, cells were inoculated in 6-well plates and collected when they reached 70% confluence. The cells were washed twice with PBS, resuspended in 100 μL of 1× binding buffer and stained with 5 μL of Annexin V-FITC and 10 μL of PI staining solution for 15 min at room temperature. Subsequently, the cells were incubated with 400 μL of 1x binding buffer at room temperature, and apoptosis was detected on a flow cytometer. The experiment was repeated three times independently.

### Assessment of apoptotic vesicles

After the cells reached 70% confluence, the culture medium and adherent cells were collected in a 2-mL centrifuge tube. After centrifugation (1500 r/min, 4 min), the supernatant was discarded and the cells were incubated with an electron microscope fixative (containing 2.5% glutaraldehyde and 100-mM phosphate) at room temperature for 30 min in the dark. Subsequently, the cell samples were sent to Wuhan Servicebio Technology CO., Ltd for embedding, sectioning and transmission electron microscopy to observe apoptotic vesicles and cell morphology.

### Assessment of mitochondrial membrane potential

Cellular mitochondrial membrane potential was evaluated using the Mitochondrial Membrane Potential Assay Kit (JC-1). Briefly, cells were inoculated in a special dish for laser confocal microscopy. When the cells reached 70% confluence, the medium was discarded and the cells were washed with PBS and incubated with 1 mL of a medium and 1 mL of JC-1 staining working solution (JC-1 [200 X]: ultrapure water: JC-1 staining buffer [5×] = 1:160:40) at 37 °C for 30 min. Subsequently, the cells were washed twice with JC-1 buffer (JC-1 staining buffer [5×]: distilled water = 1:4). Finally, 2 mL of cell culture solution was added and fluorescence was detected under laser confocal microscope. Fluorescence was detected using a laser confocal microscope, with red fluorescence indicating normal mitochondrial membrane potential and green fluorescence indicating low mitochondrial membrane potential and early stage of cell apoptosis.

### qRT-PCR

Total RNA was extracted from HCC cells using the RNAiso Plus reagent. The quality and concentration of the extracted RNA were measured on a microspectrophotometer. The extracted RNA was reverse transcribed to cDNA using the TB Green Premix Ex Taq II Kit. RT-PCR was performed on an Applied Biosystems Gene Amplification PCR instrument, and qPCR was performed on a BIO-RAD CFX96 Detection System. The mRNA expression of target genes was calculated using the 2^-ΔΔCt^ method, with β-actin being used as the negative control. The sequences of all PCR primers are provided in Supplementary Table 2. The experiment was repeated three times independently.

### Western blotting

Total proteins were extracted from HCC cells using an IP lysate (PMSF:IP = 1:100) containing the proteasome inhibitor phenylmethylsulphonyl fluoride (PMSF). After the extracted proteins were quantified using a BCA protein assay kit, they were mixed with 5× SDS-PAGE loading buffer (1:4 by volume). Equal amounts of protein samples were separated via SDS-PAGE, and the separated proteins were transferred to a polyvinylidene fluoride (PVDF) membrane. The membrane was blocked with 5% skimmed milk for 1.5 h, washed thrice with PBST and incubated with specific primary antibodies on a shaker at 4°C overnight. The following day, the membrane was incubated with horseradish peroxidase-labelled goat anti-rabbit secondary antibody at room temperature for 1 h. Thereafter, the membrane was washed thrice with PBST, and protein bands were visualised using an ultra-sensitive enhanced chemiluminescence (ECL) reagent on a high-sensitivity luminescence imaging system (Tanon 5200). The grayscale values of target bands were calculated using the Image Lab (version 6.0) software (Bio-Rad). Please refer to Supplementary Table 3 for detailed information on all antibodies, including their Cat numbers, manufacturers and dilution concentrations. The experiment was repeated three times independently.

### Co-immunoprecipitation

Co-immunoprecipitation (Co-IP) assay was performed using the Pierce™ Co-Immunoprecipitation Kit according to the manufacturer’s instructions. Total proteins were extracted from cells and quantified. Supernatants containing 2 mg of protein were incubated with 4 μg of anti-NAP1L1, anti-UBR4, anti-BIRC2 or IgG antibodies overnight at 4 °C on a tumbling shaker. After elution with magnetic beads, the recovered proteins were mixed with 5× SDS-PAGE loading buffer (1:4 by volume) and incubated in a water bath at 100°C for 10 min. Protein immunocomplexes were detected via western blotting, and anti-IgG antibody was used as the negative control.

### Immunohistochemical analysis

This study was approved by the Ethics Committee of Guizhou Medical University Hospital (NO: 2022053). A total of 20 pairs of HCC and adjacent normal tissue samples were collected from patients with HCC who underwent liver surgery. In addition, the tumour tissues of nude mice in the sh-NC and sh-NAP1L1 groups in this study were obtained from our previous study [13].

Tissue microarray (TMA) (Shanghai Tufei Biotechnology Co., Ltd) was used to detect the protein expression of BIRC2. TMA was dewaxed, hydrated, subjected to antigen retrieval, blocked with endogenous peroxidase, incubated with primary and secondary (goat anti-mouse IgG) antibodies, stained with DAB, re-stained, sealed, observed under a microscope and photographed at a single time point according to the instructions of the mouse two-step assay kit. The staining intensity was independently scored by two pathologists who were blinded to the clinical information of patients. The extent of staining, defined as the percentage of positively stained tumour cells with respect to the whole tissue area, was scored on a scale of 0–4 as follows: 0, <10%; 1, 10–25%; 2, 26–50%; 3, 50–75%; 4, >75%. The staining intensity was scored on a scale of 0–3 as follows: 0, negative; 1, weak; 2, moderate; 3, strong. The immunohistochemical score of each sample was calculated as the product of the extent of staining and the intensity of staining [13]. For statistical analysis, an immunohistochemical score of 0–6 indicated low expression and a score of 6–12 indicated high expression.

### Mass spectrometry

293 T cells were transfected with NAP1L1-overexpression or empty plasmids. After the transfection efficiency (in 293T-NAP1L1 and 293T-NC groups) was verified via qPCR, the cell samples were sent to Zhongke New Life (Zhejiang) Biotechnology Co., Ltd. for mass spectrometry. The results are shown in Additional file 3.

### Bioinformatic analysis

The BIOGRID database (https://thebiogrid.org/) was used to screen for potential biomarkers interacting with NAP1L1 and BIRC2. The UALCAN web resource (https://ualcan.path.uab.edu/index.html) was used to extract TCGA data and analyse BIRC2 expression and its relationship with survival in HCC.

### Statistical analysis

All data were analysed using the SPSS Statistics (version 24.0) software (International Business Machines Corporation, IBM, New York, USA) and expressed as the mean ± SD of at least three independent experiments. The Kolmogorov–Smirnov test was used to assess the normality of the data. Independent samples *t*-test was used to compare the data of two groups, whereas one-way ANOVA was used to compare the data of three or more groups. Survival analysis was performed using the Kaplan–Meier plotter and log-rank test. Cox proportional-hazard regression models were used to screen for independent risk factors affecting prognosis. All statistical tests were two-sided; single, double and triple asterisks were used to indicate statistical significance (^∗^*p* < 0.05; ^∗∗^*p* < 0.01; ^∗∗∗^*p* < 0.001).

### Supplementary information

Supplemental Figures and Tables

Additional file 1

Additional file 2

Additional file 3

Supplemental file_Uncropped WB

## Data Availability

All datasets generated and analysed during this study are included in this published article and its Supplementary Information files. Additional data are available from the corresponding author on reasonable request.

## References

[1] Llovet JM, Castet F, Heikenwalder M, Maini MK, Mazzaferro V, Pinato DJ (2022). Immunotherapies for hepatocellular carcinoma. Nat Rev Clin Oncol.

[2] McGlynn KA, Petrick JL, El-Serag HB (2021). Epidemiology of hepatocellular carcinoma. Hepatology.

[3] Chen Y, Chen HN, Wang K, Zhang L, Huang Z, Liu J (2019). Ketoconazole exacerbates mitophagy to induce apoptosis by downregulating cyclooxygenase-2 in hepatocellular carcinoma. J Hepatol.

[4] Tang W, Chen Z, Zhang W, Cheng Y, Zhang B, Wu F (2020). The mechanisms of sorafenib resistance in hepatocellular carcinoma: theoretical basis and therapeutic aspects. Signal Transduct Target Ther.

[5] Xie X, Wang X, Liao W, Fei R, Wu N, Cong X (2019). PPPDE1 promotes hepatocellular carcinoma development by negatively regulate p53 and apoptosis. Apoptosis.

[6] Holgado E, Perez JM, Wren A, Cortes J, Gomez-Pinillos A (2015). Influencing cancer treatment. Lancet Oncol.

[7] Mohammad RM, Muqbil I, Lowe L, Yedjou C, Hsu HY, Lin LT (2015). Broad targeting of resistance to apoptosis in cancer. Semin Cancer Biol.

[8] Sperandio RC, Pestana RC, Miyamura BV, Kaseb AO (2022). Hepatocellular carcinoma immunotherapy. Annu Rev Med.

[9] Lin Z, Wan AH, Sun L, Liang H, Niu Y, Deng Y (2023). N6-methyladenosine demethylase FTO enhances chemo-resistance in colorectal cancer through SIVA1-mediated apoptosis. Mol Ther.

[10] Xu D, Zhao H, Jin M, Zhu H, Shan B, Geng J (2020). Modulating TRADD to restore cellular homeostasis and inhibit apoptosis. Nature.

[11] Roberts JZ, Crawford N, Longley DB (2022). The role of ubiquitination in apoptosis and necroptosis. Cell Death Differ.

[12] Lee JY, Lake RJ, Kirk J, Bohr VA, Fan HY, Hohng S (2017). NAP1L1 accelerates activation and decreases pausing to enhance nucleosome remodeling by CSB. Nucleic Acids Res.

[13] Zhang YW, Chen Q, Li B, Li HY, Zhao XK, Xiao YY (2021). NAP1L1 functions as a tumor promoter via recruiting hepatoma-derived growth factor/c-Jun signal in hepatocellular carcinoma. Front Cell Dev Biol.

[14] Liu S, Zhang Y, Cui S, Song D, Li B, Chen Q (2021). NAP1L1 interacts with hepatoma-derived growth factor to recruit c-Jun inducing breast cancer growth. Cancer Cell Int.

[15] Le Y, Kan A, Li QJ, He MK, Chen HL, Shi M (2019). NAP1L1 is a prognostic biomarker and contribute to doxorubicin chemotherapy resistance in human hepatocellular carcinoma. Cancer Cell Int.

[16] Liang X, Tang Z, Zhang Y, Sun Y, Wang J (2022). NAP1L1 promotes the growth of colon cancer by activating HDGF/DDX5. Acta Biochim Biophys Sin.

[17] Tanaka T, Hozumi Y, Iino M, Goto K (2017). NAP1L1 regulates NF-kappaB signaling pathway acting on anti-apoptotic Mcl-1 gene expression. Biochim Biophys Acta Mol Cell Res.

[18] Li T, Niu Z, Yu T, Li J, Lu X, Huang M (2023). Nucleosome assembly protein 1 like 1 (NAP1L1) promotes cardiac fibrosis by inhibiting YAP1 ubiquitination and degradation. MedComm.

[19] Wang L, Du F, Wang X (2008). TNF-alpha induces two distinct caspase-8 activation pathways. Cell.

[20] Samanta D, Huang TY, Shah R, Yang Y, Pan F, Semenza GL (2020). BIRC2 expression impairs anti-cancer immunity and immunotherapy efficacy. Cell Rep.

[21] Bertrand MJ, Milutinovic S, Dickson KM, Ho WC, Boudreault A, Durkin J (2008). cIAP1 and cIAP2 facilitate cancer cell survival by functioning as E3 ligases that promote RIP1 ubiquitination. Mol Cell.

[22] Majorini MT, Manenti G, Mano M, De Cecco L, Conti A, Pinciroli P (2018). cIAP1 regulates the EGFR/Snai2 axis in triple-negative breast cancer cells. Cell Death Differ.

[23] Bai L, Smith DC, Wang S (2014). Small-molecule SMAC mimetics as new cancer therapeutics. Pharmacol Ther.

[24] Cheng S, Jiang X, Ding C, Du C, Owusu-Ansah KG, Weng X (2016). Expression and Critical Role of Interleukin enhancer binding factor 2 in hepatocellular carcinoma. Int J Mol Sci.

[25] Fan L, Sun G, Ma T, Zhong F, Wei W (2013). Melatonin overcomes apoptosis resistance in human hepatocellular carcinoma by targeting survivin and XIAP. J Pineal Res.

[26] Ding J, Qin D, Zhang Y, Li Q, Li Y, Li J (2020). SMAC mimetic birinapant inhibits hepatocellular carcinoma growth by activating the cIAP1/TRAF3 signaling pathway. Mol Med Rep.

[27] Ma O, Cai WW, Zender L, Dayaram T, Shen J, Herron AJ (2009). MMP13, Birc2 (cIAP1), and Birc3 (cIAP2), amplified on chromosome 9, collaborate with p53 deficiency in mouse osteosarcoma progression. Cancer Res.

[28] Yang X, Zhang Y, Xue Z, Hu Y, Zhou W, Xue Z (2022). TRIM56 promotes malignant progression of glioblastoma by stabilizing cIAP1 protein. J Exp Clin Cancer Res.

[29] Hunt LC, Schadeberg B, Stover J, Haugen B, Pagala V, Wang YD (2021). Antagonistic control of myofiber size and muscle protein quality control by the ubiquitin ligase UBR4 during aging. Nat Commun.

[30] Kim JG, Shin HC, Seo T, Nawale L, Han G, Kim BY (2021). Signaling Pathways Regulated by UBR Box-Containing E3 Ligases. Int J Mol Sci.

[31] Tasaki T, Kim ST, Zakrzewska A, Lee BE, Kang MJ, Yoo YD (2013). UBR box N-recognin-4 (UBR4), an N-recognin of the N-end rule pathway, and its role in yolk sac vascular development and autophagy. Proc Natl Acad Sci USA.

[32] Tang D, Sandoval W, Lam C, Haley B, Liu P, Xue D (2020). UBR E3 ligases and the PDIA3 protease control degradation of unfolded antibody heavy chain by ERAD. J Cell Biol.

[33] Feng L, Liu T, Shi J, Wang Y, Yang Y, Xiao W (2023). Circ-UBR4 regulates the proliferation, migration, inflammation, and apoptosis in ox-LDL-induced vascular smooth muscle cells via miR-515-5p/IGF2 axis. Open Med.

[34] Hegazi S, Cheng AH, Krupp JJ, Tasaki T, Liu J, Szulc DA (2022). UBR4/POE facilitates secretory trafficking to maintain circadian clock synchrony. Nat Commun.

[35] Yu X, Yin H, Peng H, Lu G, Liu Z, Dang Z (2019). OPFRs and BFRs induced A549 cell apoptosis by caspase-dependent mitochondrial pathway. Chemosphere.

[36] Yang C, Zhang H, Zhang L, Zhu AX, Bernards R, Qin W (2023). Evolving therapeutic landscape of advanced hepatocellular carcinoma. Nat Rev Gastroenterol Hepatol.

[37] Toh MR, Wong EYT, Wong SH, Ng AWT, Loo LH, Chow PK (2023). Global Epidemiology and Genetics of Hepatocellular Carcinoma. Gastroenterology.

[38] Rimassa L, Finn RS, Sangro B (2023). Combination immunotherapy for hepatocellular carcinoma. J Hepatol.

[39] Brown ZJ, Tsilimigras DI, Ruff SM, Mohseni A, Kamel IR, Cloyd JM (2023). Management of Hepatocellular Carcinoma: A Review. JAMA Surg.

[40] Huang Y, Xiang B, Liu Y, Wang Y, Kan H (2022). Corrigendum to “LncRNA CDKN2B-AS1 promotes tumor growth and metastasis of human hepatocellular carcinoma by targeting let-7c-5p/NAP1L1 axis” [Cancer Lett. 2018 (Nov 28) 437:56-66]. Cancer Lett.

[41] Liu Y, Li X, Zhang Y, Tang Y, Fang W, Liu X (2021). NAP1L1 targeting suppresses the proliferation of nasopharyngeal carcinoma. Biomed Pharmacother.

[42] Gan H, Xu X, Bai Y (2022). Trametes robiniophila represses angiogenesis and tumor growth of lung cancer via strengthening let-7d-5p and targeting NAP1L1. Bioengineered.

[43] Singh A, Choudhury SD, Singh P, Kaushal S, Sharma A (2023). Disruption in networking of KCMF1 linked ubiquitin ligase impairs autophagy in CD8(+) memory T cells of patients with renal cell carcinoma. Cancer Lett.

[44] Rothe M, Pan MG, Henzel WJ, Ayres TM, Goeddel DV (1995). The TNFR2-TRAF signaling complex contains two novel proteins related to baculoviral inhibitor of apoptosis proteins. Cell.

[45] Shu HB, Takeuchi M, Goeddel DV (1996). The tumor necrosis factor receptor 2 signal transducers TRAF2 and c-IAP1 are components of the tumor necrosis factor receptor 1 signaling complex. Proc Natl Acad Sci USA.

[46] Deveraux QL, Roy N, Stennicke HR, Van Arsdale T, Zhou Q, Srinivasula SM (1998). IAPs block apoptotic events induced by caspase-8 and cytochrome c by direct inhibition of distinct caspases. EMBO J.

[47] Wang CY, Mayo MW, Korneluk RG, Goeddel DV, Baldwin AS (1998). Jr. NF-kappaB antiapoptosis: induction of TRAF1 and TRAF2 and c-IAP1 and c-IAP2 to suppress caspase-8 activation. Science.

[48] Wu P, Shi KJ, An JJ, Ci YL, Li F, Hui KY (2014). The LEF1/CYLD axis and cIAPs regulate RIP1 deubiquitination and trigger apoptosis in selenite-treated colorectal cancer cells. Cell Death Dis.

[49] Dittmann J, Haydn T, Metzger P, Ward GA, Boerries M, Vogler M (2020). Next-generation hypomethylating agent SGI-110 primes acute myeloid leukemia cells to IAP antagonist by activating extrinsic and intrinsic apoptosis pathways. Cell Death Differ.

[50] Dondelinger Y, Aguileta MA, Goossens V, Dubuisson C, Grootjans S, Dejardin E (2013). RIPK3 contributes to TNFR1-mediated RIPK1 kinase-dependent apoptosis in conditions of cIAP1/2 depletion or TAK1 kinase inhibition. Cell Death Differ.

[51] Li M, Wei Y, Liu Y, Wei J, Zhou X, Duan Y (2023). BRD7 inhibits enhancer activity and expression of BIRC2 to suppress tumor growth and metastasis in nasopharyngeal carcinoma. Cell Death Dis.

[52] Su W, Jiang X, Chen M, Huang M, Tang N, Wang X (2019). cIAP1 promotes proliferation and migration and prevents apoptosis in gallbladder cancer in vitro. Biosci Rep.

[53] Wang A, Liu J, Yang Y, Chen Z, Gao C, Wang Z (2021). Shikonin promotes ubiquitination and degradation of cIAP1/2-mediated apoptosis and necrosis in triple negative breast cancer cells. Chin Med.

[54] Schwarzenbach C, Tatsch L, Brandstetter Vilar J, Rasenberger B, Beltzig L, Kaina B (2021). Targeting c-IAP1, c-IAP2, and Bcl-2 eliminates senescent glioblastoma cells following temozolomide treatment. Cancers.

[55] Lopez J, John SW, Tenev T, Rautureau GJ, Hinds MG, Francalanci F (2011). CARD-mediated autoinhibition of cIAP1’s E3 ligase activity suppresses cell proliferation and migration. Mol Cell.

[56] Dumetier B, Zadoroznyj A, Berthelet J, Causse S, Allegre J, Bourgeois P (2023). cIAP1/TRAF2 interplay promotes tumor growth through the activation of STAT3. Oncogene.

[57] Spitz AZ, Gavathiotis E (2022). Physiological and pharmacological modulation of BAX. Trends Pharmacol Sci.

[58] Whelan RS, Konstantinidis K, Wei AC, Chen Y, Reyna DE, Jha S (2012). Bax regulates primary necrosis through mitochondrial dynamics. Proc Natl Acad Sci USA.

