# Supplementary material for: NAP1L1 regulates BIRC2 ubiquitination modification via E3 ubiquitin ligase UBR4 and hence determines hepatocellular carcinoma progression
Source: Cell Death Discov. 2024 Mar 27;10:154. doi: 10.1038/s41420-024-01927-2 (PMC10973488; doi:10.1038/s41420-024-01927-2)
Supplement: Supplementary file 1 — Supplemental Figures and Tables [file 41420_2024_1927_MOESM1_ESM.pdf]

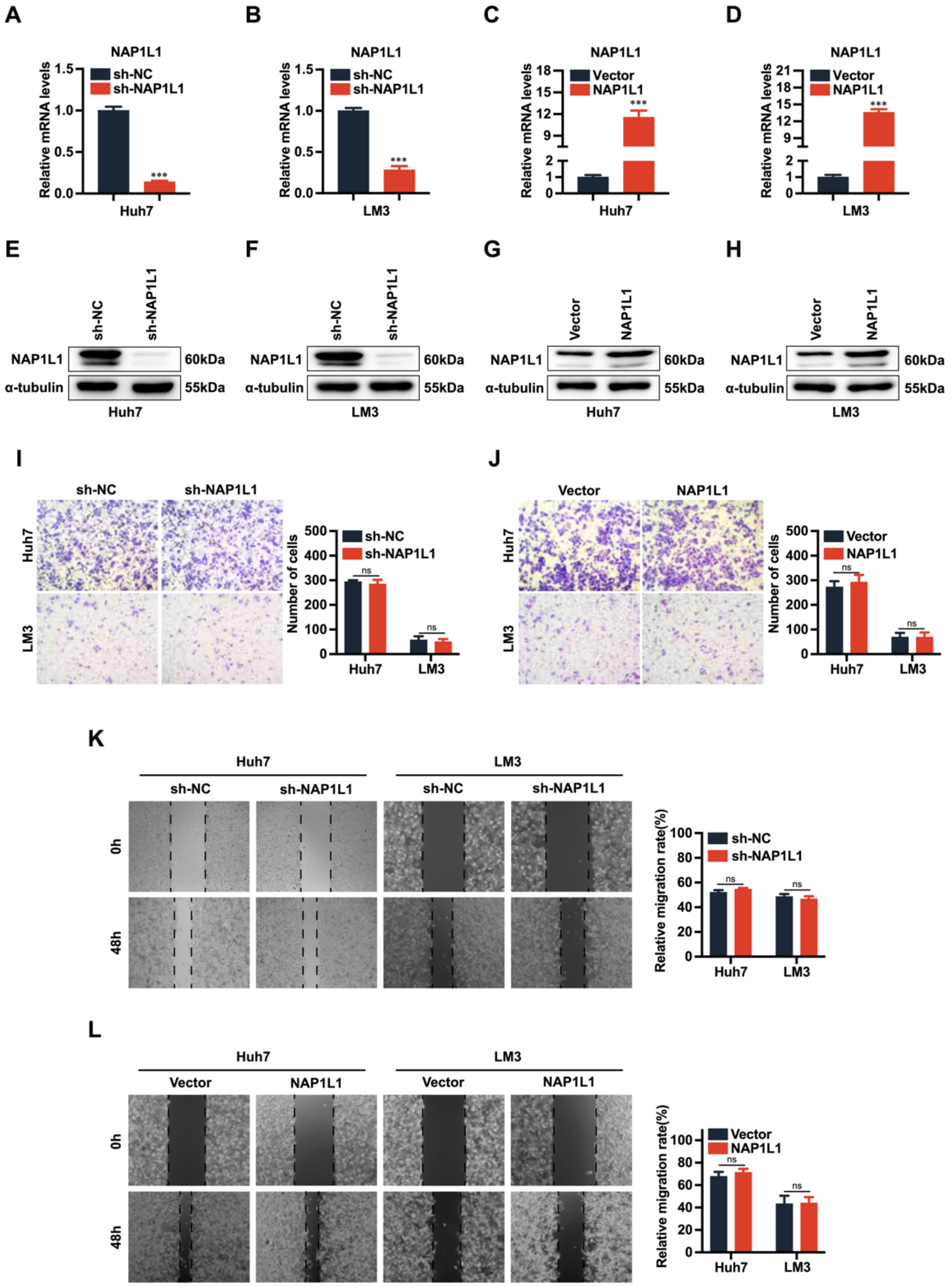

Figure S1 NAP1L1 does not affect the migratory and invasive abilities of HCC cells.

(A–D) qRT-PCR was performed to evaluate the mRNA expression of NAP1L1 in Huh7 and LM3 cells after knockdown/overexpression of NAP1L1. (E–H) Western blotting was performed to evaluate the protein expression of NAP1L1 in Huh7 and LM3 cells after knockdown/overexpression of NAP1L1. (I–J) Transwell assay was performed to assess the invasive ability of Huh7 and LM3 cells after knockdown/overexpression of NAP1L1. (K–L) Wound healing assay was performed to assess

the migratory ability of Huh7 and LM3 cells after knockdown/overexpression of NAP1L1. Data are representative of three independent experiments and are expressed as the mean ± SD (*, $p < 0.05$ versus control; **, $p < 0.01$; ***, $p < 0.001$).

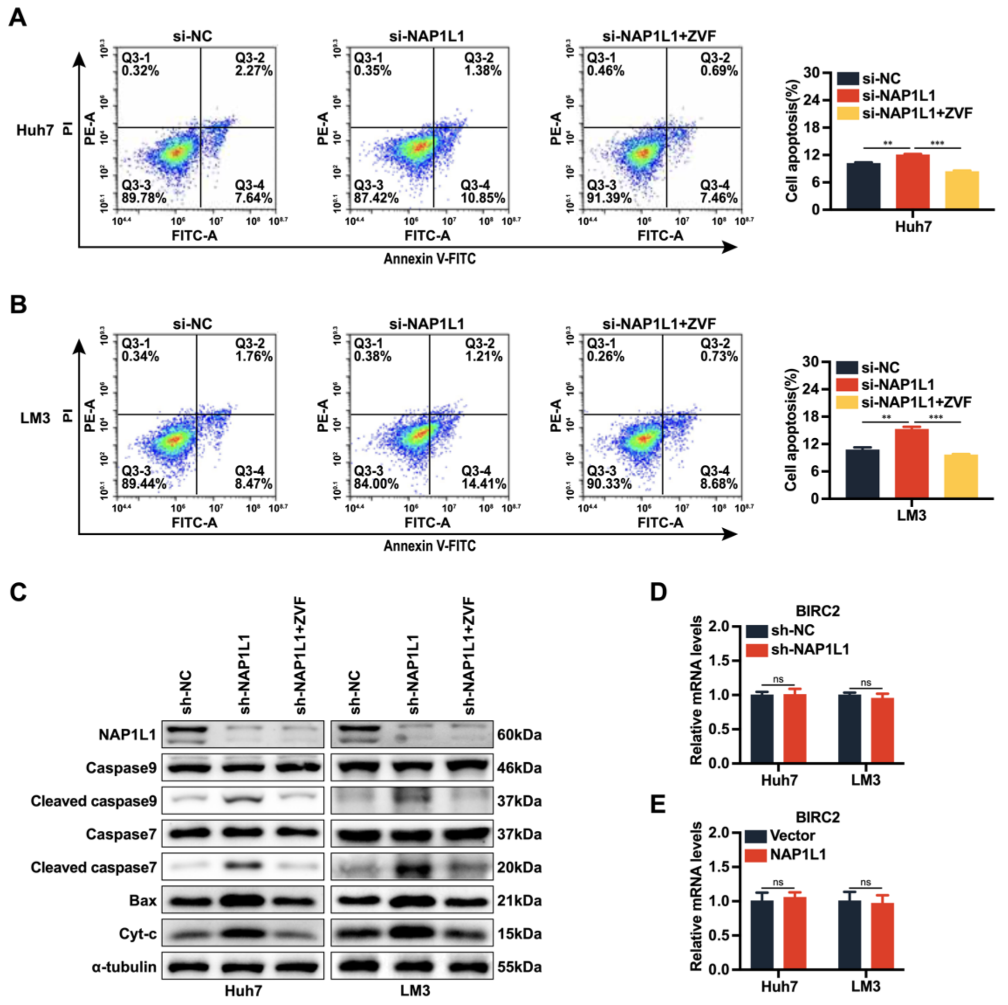

Figure S2 NAP1L1 affects hepatocellular carcinoma cell apoptosis through the caspase pathway.

(A–B) Flow cytometry was performed to evaluate the apoptosis levels of Huh7 and LM3 cells treated with the apoptosis inhibitor (ZVF) and/or transfected with siNAP1L1. (C) Western blotting was performed to evaluate the expression of apoptosis-related proteins in Huh7 and LM3 cells treated with ZVF and/or transfected with siNAP1L1. (D–E) qRT-PCR was performed to assess the mRNA expression of BIRC2 in Huh7 and LM3 cells after knockdown/overexpression of NAP1L1. Data are representative of three independent experiments and are expressed as the mean ± SD (*, $p < 0.05$

versus control; **, $p < 0.01$; ***, $p < 0.001$).

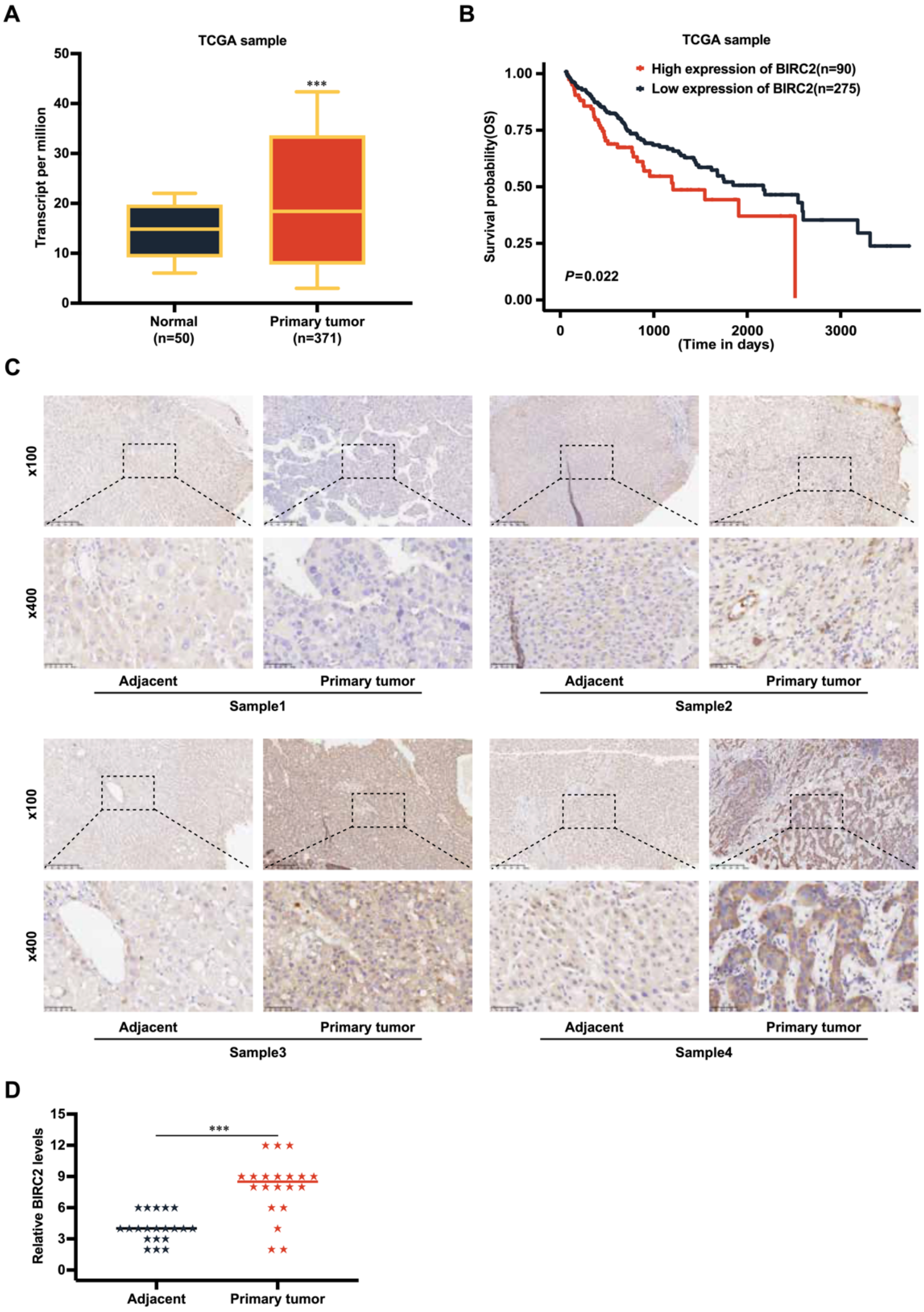

Figure S3 BIRC2 is significantly upregulated in HCC tissues and is associated with a poor prognosis.

(A) mRNA expression of BIRC2 in HCC tissues and adjacent paracancerous tissues in TCGA cohort (https://ualcan.path.uab.edu/index.html). (B) Kaplan–Meier analysis of overall survival based on BIRC2

expression. (C) Immunohistochemical staining was performed to assess BIRC2 expression in HCC and paracancerous tissues (×100 visual field, scale bar = 250 μm; ×400, scale bar = 50 μm). (D) Immunohistochemical scoring of 20 pairs of clinical HCC and adjacent normal tissues (*, $p < 0.05$ versus control; **, $p < 0.01$; ***, $p < 0.001$).

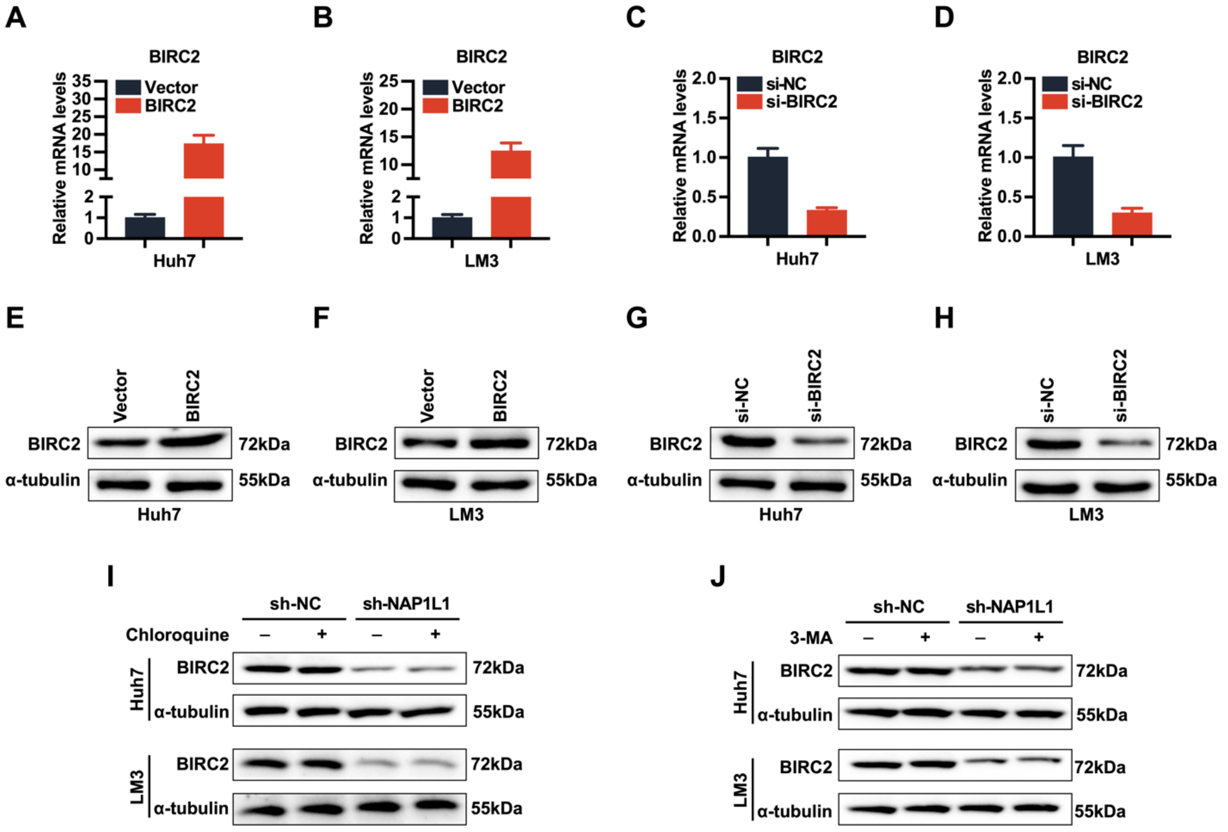

Figure S4 Validation of the efficiency of knockdown/overexpression of BIRC2.

(A–D) qRT-PCR was performed to evaluate the mRNA expression of BIRC2 in Huh7 and LM3 cells after knockdown/overexpression of BIRC2. (E–H) Western blotting was performed to evaluate the protein expression of BIRC2 in Huh7 and LM3 cells after knockdown/overexpression of BIRC2. (I–J) Protein expression of BIRC2 in Huh7/LM3 cells transfected with shNAP1L1 and treated with chloroquine and 3-MA as indicated. Data are representative of three independent experiments and are expressed as the mean ± SD (*, $p < 0.05$ versus control; **, $p < 0.01$; ***, $p < 0.001$).

Table S1. Sequences for gene transient and stable knockdown or overexpression plasmids.

Gene	Accession	NO.	Target Seq
NAP1L1	stB0007176A	st-h-NAP1L1_001	GAAGTATGCTGTTCTCTAT
NAP1L1	NM_004537	NAP1L1-RNAi (78814-1)	GCCAAGATTGAAGATGAGAAA
NAP1L1	NM_004537	NAP1L1(62885-1)-p1	ATGGCAGACATTGACAACAAAGAACAGTCTGAACTTGATCAAGATTTGGA<br>TGATGTTGAAGAAGTAGAAGAAGAGGAAACTGGTGAAGAAACAAAACT<br>CAAAGCACGTCAGCTAACTGTTCAGATGATGCAAAATCCTCAGATTCTTG<br>CAGCCCTTCAAGAAAGACTTGATGGTCTGGTAGAAACACCAACAGGATAC<br>ATTGAAAGCCTGCCTAGGGTAGTTAAAAGACGAGTGAATGCTCTCAAAAA<br>CCTGCAAGTTAAATGTGCACAGATAGAAGCCAAATTCTATGAGGAAGTTC<br>ATGATCTTGAAAGGAAGTATGCTGTTCTCTATCAGCCTCTATTTGATAAGCG<br>ATTTGAAATTATTAATGCAATTTATGAACCTACGGAAGAAGAATGTGAATG<br>GAAACCAGATGAAGAAGATGAGATTTCGGAGGAATTGAAAGAAAAGGCC<br>AAGATTGAAGATGAGAAAAAGGATGAAGAAAAAGAAGACCCCAAAGGA<br>ATTCCTGAATTTTGGTTAACTGTTTTTAAGAATGTTGACTTGCTCAGTGATA<br>TGGTTCAGGAACACGATGAACCTATTCTGAAGCACTTGAAAGATATTAAA<br>GTGAAGTTCTCAGATGCTGGCCAGCCTATGAGTTTTGTCTTAGAATTTCAC<br>TTTGAACCCAATGAATATTTTACAAATGAAGTGCTGACAAAGACATACAG<br>GATGAGGTCAGAACCAGATGATTCTGATCCCTTTTCTTTTGATGGACCAGA<br>AATTATGGGTTGTACAGGATGCCAGATAGATTGGAAAAAAGGAAAGAATG<br>TCACTTTGAAAACTATTAAGAAGAAGCAGAAACACAAGGGACGTGGGAC<br>AGTTCGTACTGTGACTAAAACAGTTTCCAATGACTCTTTCTTTAACTTTTT<br>TGCCCCTCCTGAAGTTCCTGAGAGTGGAGATCTGGATGATGATGCTGAAG<br>CTATCCTTGCTGCAGACTTCGAAATTGGTCACTTTTTACGTGAGCGTATAAT<br>CCCAAGATCAGTGTTATATTTTACTGGAGAAGCTATTGAAGATGATGATGA<br>TGATTATGATGAAGAAGGTGAAGAAGCGGATGAGGAAGGGGAAGAAGAA<br>GGAGATGAGGAAAATGATCCAGACTATGACCCAAAGAAGGATCAAAACC<br>CAGCAGAGTGCAAGCAGCAG
UBR4	stB0000915A	st-h-UBR4_001	CAAGTAGCCTGCAGTATGA
BIRC2	stB0002397A	st-h-BIRC2_001	GGATCCACCTCTAAGAATA

BIRC2	NM_001166	pCDH-CMV-BIRC2-3Flag-tRFP-F2A-Neo	ATGCACAAAACTGCCTCCCAAAGACTTTTCCCAGGTCCCTCGTATCAAAA<br>CATTAAGAGTATAATGGAAGATAGCACGATCTTGTCAGATTGGACAAACA<br>GCAACAAACAAAAAATGAAGTATGACTTTTCCTGTGAACTCTACAGAATG<br>TCTACATATTCAACTTTCCCCGCCGGGGTGCCTGTCTCAGAAAGGAGTCTT<br>GCTCGTGCTGGTTTTTATTATACTGGTGTGAATGACAAGGTCAAATGCTTC<br>TGTTGTGGCCTGATGCTGGATAACTGGAAACTAGGAGACAGTCCTATTCA<br>AAAGCATAAACAGCTATATCCTAGCTGTAGCTTTATTCAGAATCTGGTTTC<br>AGCTAGTCTGGGATCCACCTCTAAGAATACGTCTCCAATGAGAAACAGTT<br>TTGCACATTCATTATCTCCCACCTTGGAACATAGTAGCTTGTTCAGTGGTTC<br>TTACTCCAGCCTTTCTCCAAACCCTCTTAATTCTAGAGCAGTTGAAGACAT<br>CTCTTCATCGAGGACTAACCCCTACAGTTATGCAATGAGTACTGAAGAAG<br>CCAGATTTCTTACCTACCATATGTGGCCATTAACTTTTTTGTCACCATCAGA<br>ATTGGCAAGAGCTGGTTTTTATTATATAGGACCTGGAGATAGGGTAGCCTG<br>CTTTGCCTGTGGTGGGAAGCTCAGTAACTGGGAACCAAAGGATGATGCTA<br>TGTCAGAACACCGGAGGCATTTTCCCAACTGTCCATTTTTGGAAAATTCTC<br>TAGAAACTCTGAGGTTTAGCATTTCAAATCTGAGCATGCAGACACATGCA<br>GCTCGAATGAGAACATTTATGTACTGGCCATCTAGTGTTCCAGTTCAGCCT<br>GAGCAGCTTGCAAGTGCTGGTTTTTATTATGTGGGTCGCAATGATGATGTC<br>AAATGCTTTTGTTGTGATGGTGGCTTGAGGTGTTGGGAATCTGGAGATGAT<br>CCATGGGTAGAACATGCCAAGTGGTTTCCAAGGTGTGAGTTCTTGATACG<br>AATGAAAGGCCAAGAGTTTGTTGATGAGATTCAAGGTAGATATCCTCATCT<br>TCTTGAACAGCTGTTGTCAACTTCAGATACCACTGGAGAAGAAAATGCTG<br>ACCCACCAATTATTCATTTTGGACCTGGAGAAAGTTCTTCAGAAGATGCTG<br>TCATGATGAATACACCTGTGGTTAAATCTGCCTTGGAAATGGGCTTTAATA<br>GAGACCTGGTGAAACAAACAGTTCAAAGTAAAATCCTGACAACTGGAGA<br>GAACTATAAAACAGTTAATGATATTGTGTCAGCACTTCTTAATGCTGAAGA<br>TGAAAAAAGAGAAGAGGAGAAGGAAAAACAAGCTGAAGAAATGGCATC<br>AGATGATTTGTCATTAATTCGGAAGAACAGAATGGCTCTCTTTCAACAATT<br>GACATGTGTGCTTCCTATCCTGGATAATCTTTTAAAGGCCAATGTAATTAAT<br>AAACAGGAACATGATATTATTAAACAAAAAACACAGATACCTTTACAAGC<br>GAGAGAACTGATTGATACCATTTTGGTTAAAGGAAATGCTGCGGCCAACA

	TCTTCAAAAACTGTCTAAAAGAAATTGACTCTACATTGTATAAGAACTTAT<br>TTGTGGATAAGAATATGAAGTATATTCCAACAGAAGATGTTTCAGGTCTGT<br>CACTGGAAGAACAATTGAGGAGGTTGCAAGAAGAACGAACTTGTAAAGT<br>GTGTATGGACAAAGAAGTTTCTGTTGTATTTATTCCTTGTGGTCATCTGGTA<br>GTATGCCAGGAATGTGCCCCTTCTCTAAGAAAATGCCCTATTTGCAGGGGT<br>ATAATCAAGGGTACTGTTCGTACATTTCTCTCT

Table S2. Primers used in this study.

Primer's name	Directional	Sequence (5'-3')
NAP1L1	Forward	TTTGCCCCTCCTGAAGTTCC
	Reverse	CCCAACACAACTTGAGACATCC
BIRC2	Forward	GCCATCTAGTGTTCCAGTTCAGC
	Reverse	CACCTCAAGCCACCATCACAAC
β-actin	Forward	ACAGAGCCTCGCCTTTGCC
	Reverse	GATATCATCATCCATGGTGAGCTGG

Table S3. List of antibodies used for WB, Co-IP and IHC staining.

Antibodies	Cat.No	Company	Species	Dulution
NAP1L1	14898-1-AP	Proteintech	Rabbit	1:1000(WB); 1:10(Co-IP); 1:150(IHC)
BIRC2	AB108361	Abcam	Rabbit	1:5000(WB); 1:100(IHC)
BIRC2	mAb#7065	Cell Sigaling	Rabbit	1:10(Co-IP)
UBR4	15966-1-AP	Proteintech	Rabbit	1:1000(WB); 1:10(Co-IP)
Caspase9<br>Cleaved caspase9	10380-1-AP	Proteintech	Rabbit	1:600(WB); 1:100(IHC)
Caspase7<br>Cleaved caspase7	27155-1-AP	Proteintech	Rabbit	1:1000(WB)
Bax	50599-2-lg	Proteintech	Rabbit	1:5000(WB)
Cytochrome c	10993-1-AP	Proteintech	Rabbit	1:3000(WB)
α-tubulin	66031-1-lg	Bioworld	Rabbit	1:10000(WB)
