# Supplementary material for: NAP1L1 regulates BIRC2 ubiquitination modification via E3 ubiquitin ligase UBR4 and hence determines hepatocellular carcinoma progression
Source: Cell Death Discov. 2024 Mar 27;10:154. doi: 10.1038/s41420-024-01927-2 (PMC10973488; doi:10.1038/s41420-024-01927-2)
Supplement: Supplementary file 5 — Supplemental file_Uncropped WB [file 41420_2024_1927_MOESM5_ESM.pdf]

Full and uncropped western blot for Figure 2A

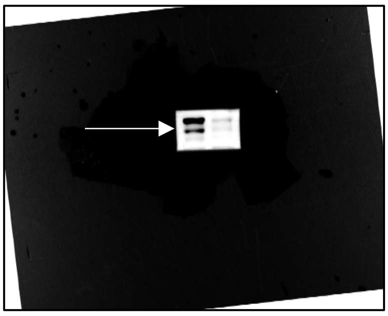
NAP1L1(Huh7)

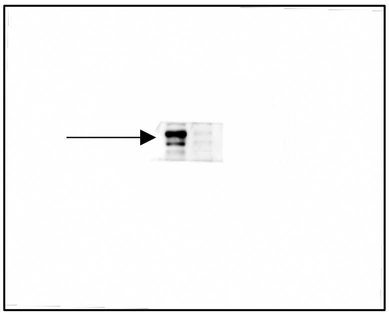
NAP1L1(LM3)

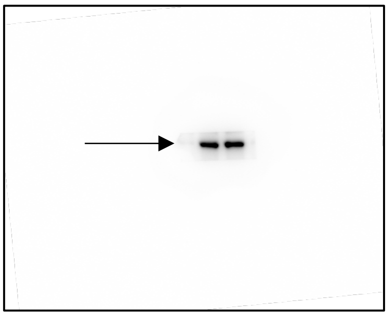
Caspase9(Huh7)

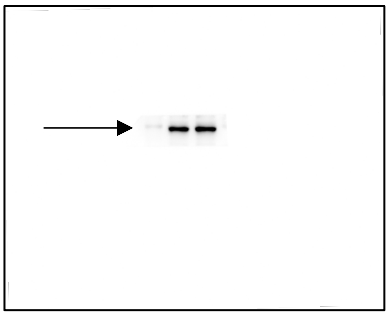
Caspase9(LM3)

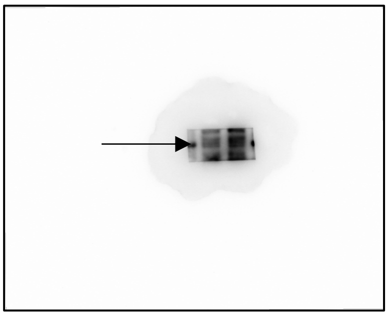
Cleaved caspase9(Huh7)

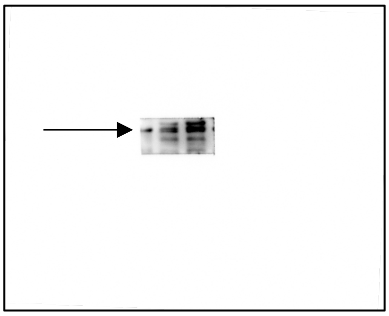
Cleaved caspase9(LM3)

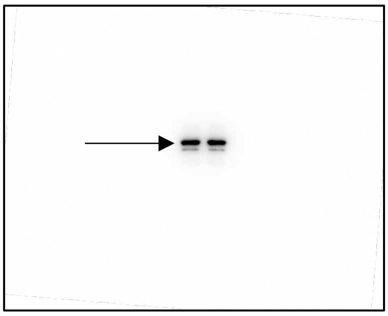
Caspase7(Huh7)

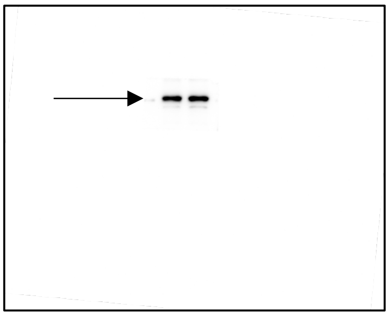
Caspase7(LM3)

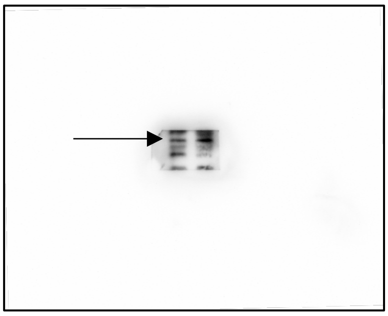
Cleaved caspase7(Huh7)

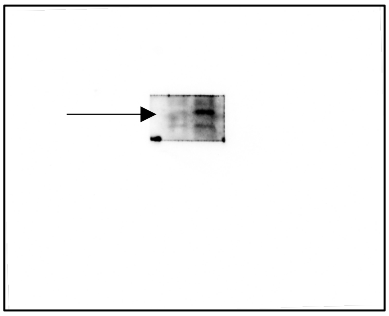
Cleaved caspase7(LM3)

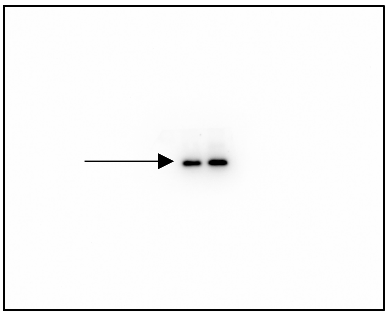

Bax(Huh7)

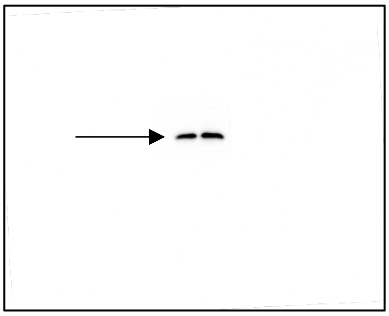

Bax(LM3)

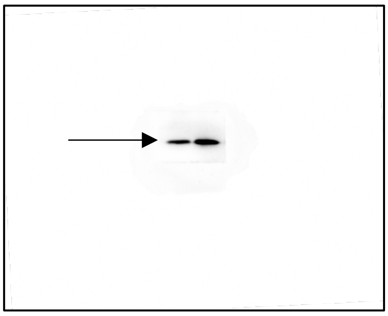

Cyt-c(Huh7)

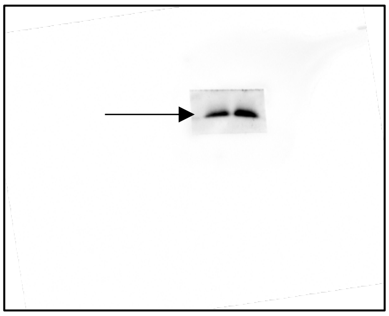

Cyt-c(LM3)

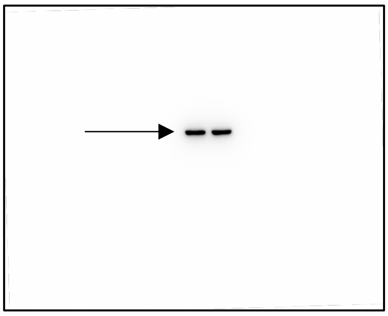

α-tubulin(Huh7)

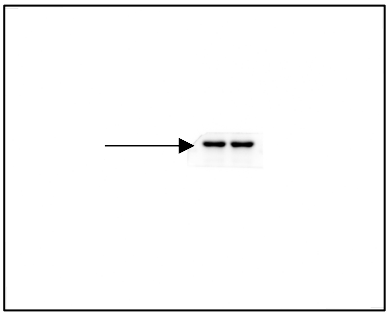

α-tubulin(LM3)

Full and uncropped western blot for Figure 2B

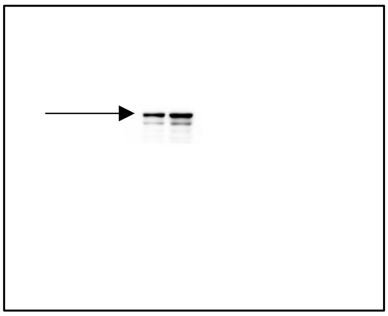

NAP1L1(Huh7)

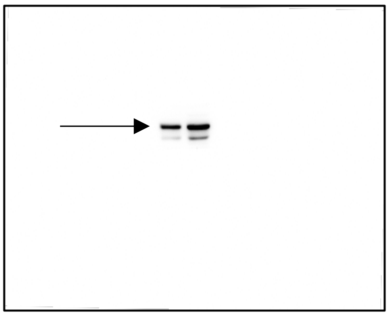

NAP1L1(LM3)

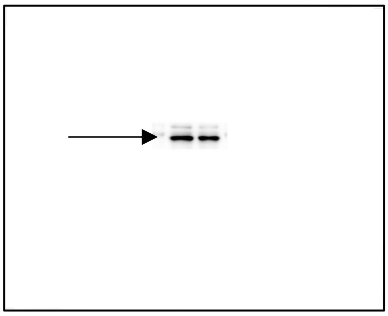

Caspase9(Huh7)

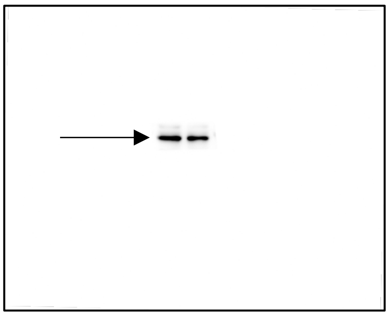

Caspase9(LM3)

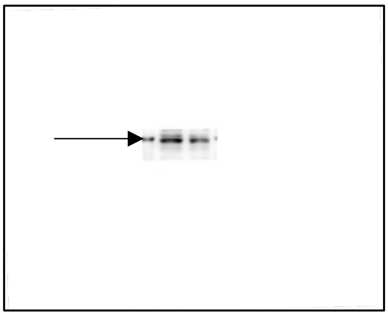

Cleaved caspase9(Huh7)

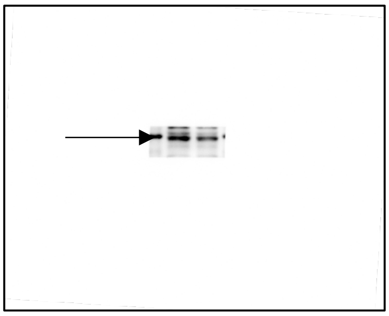

Cleaved caspase9(LM3)

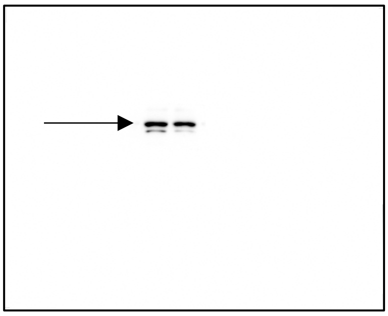

Caspase7(Huh7)

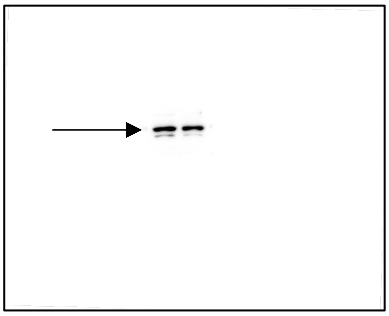

Caspase7(LM3)

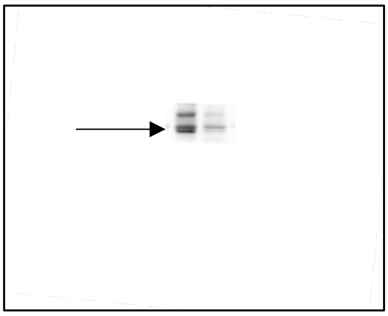

Cleaved caspase7(Huh7)

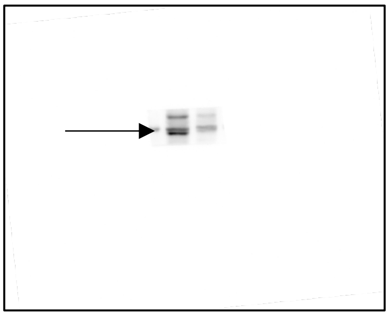

Cleaved caspase7(LM3)

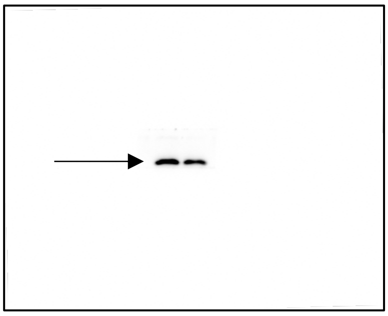

Bax(Huh7)

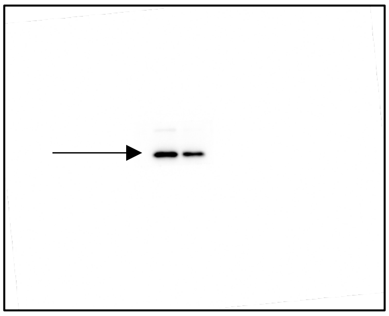

Bax(LM3)

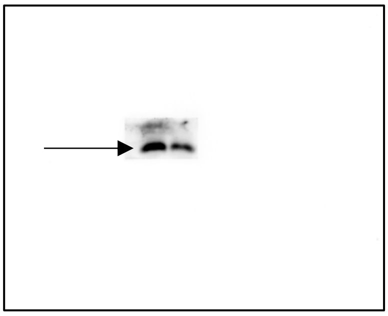

Cyt-c(Huh7)

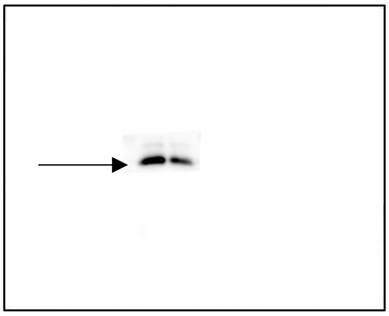

Cyt-c(LM3)

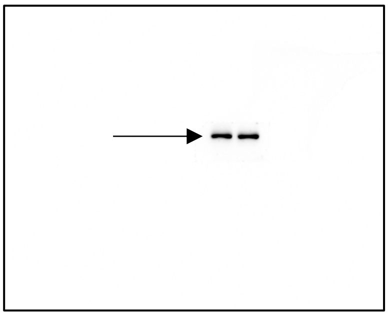

α-tubulin(Huh7)

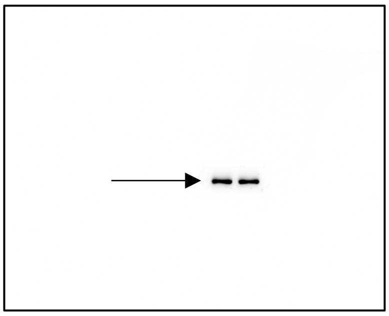

α-tubulin(LM3)

Full and uncropped western blot for Figure 2E

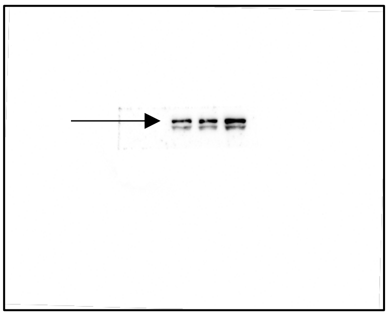

NAP1L1(Huh7)

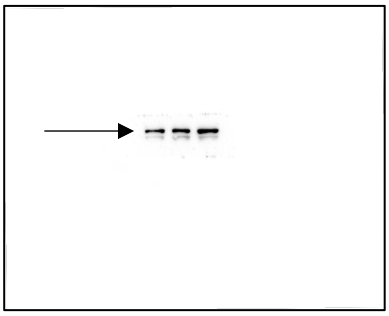

NAP1L1(LM3)

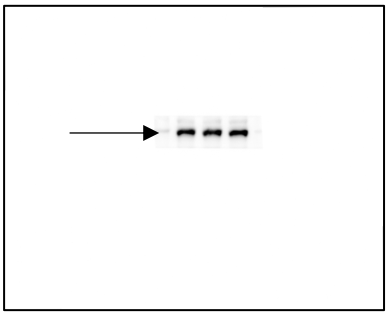

Caspase9(Huh7)

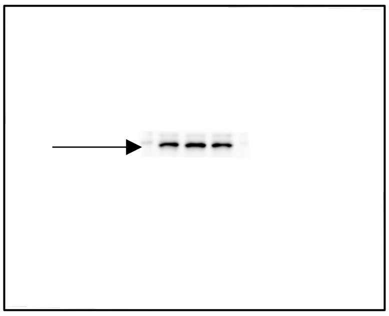

Caspase9(LM3)

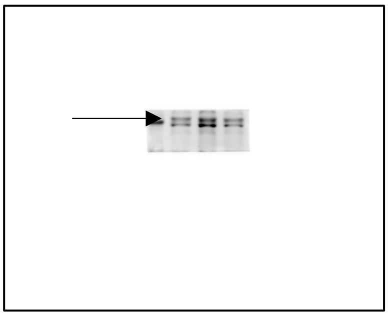

Cleaved caspase9(Huh7)

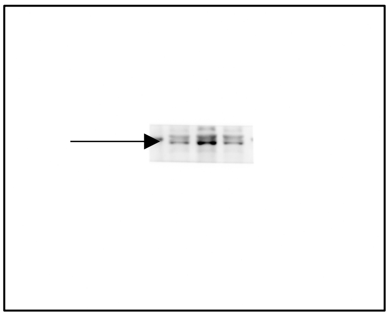

Cleaved caspase9(LM3)

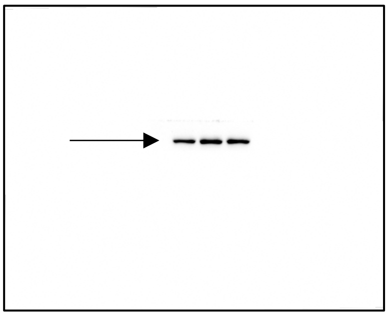

Caspase7(Huh7)

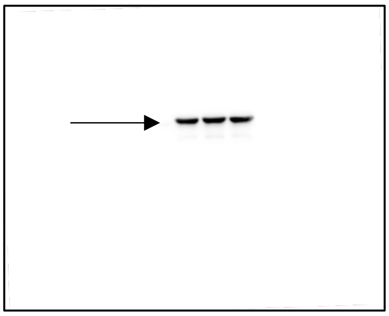

Caspase7(LM3)

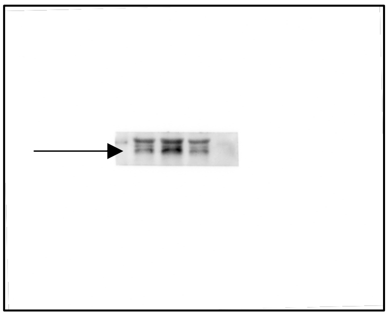

Cleaved caspase7(Huh7)

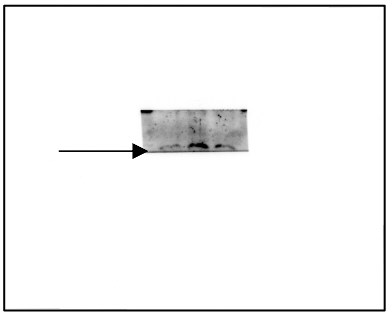

Cleaved caspase7(LM3)

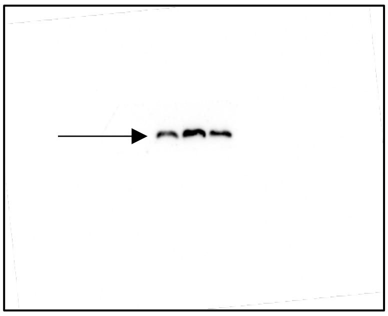

Bax(Huh7)

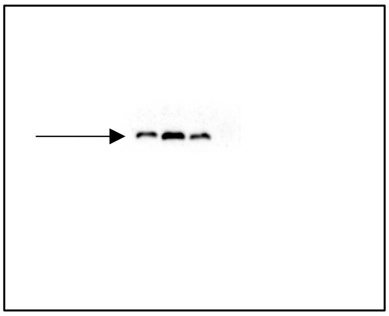

Bax(LM3)

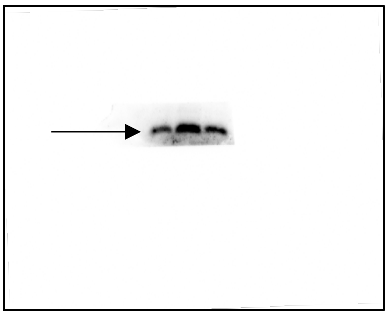

Cyt-c(Huh7)

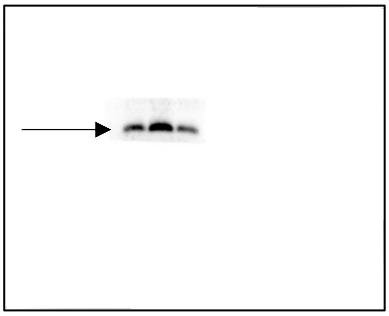

Cyt-c(LM3)

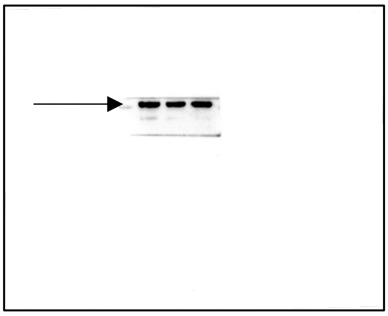

α-tubulin(Huh7)

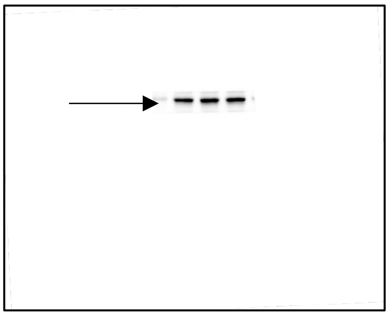

α-tubulin(LM3)

Full and uncropped western blot for Figure 2F

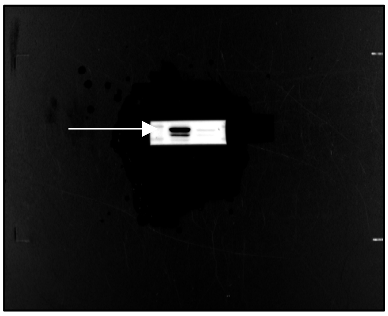

NAP1L1(Huh7)

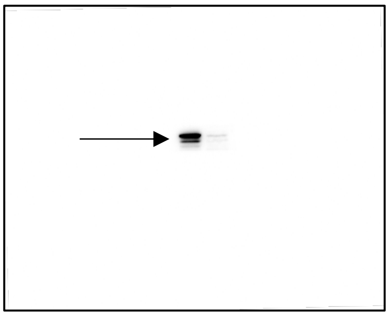

NAP1L1(LM3)

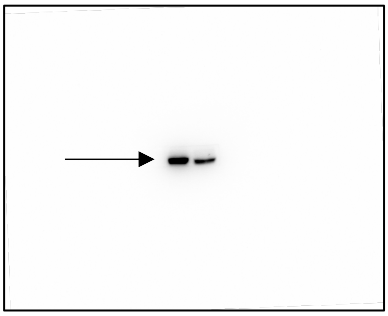

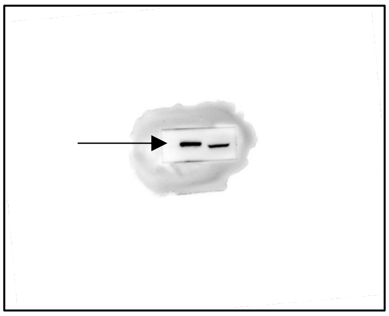

BIRC2(Huh7) BIRC2(LM3)

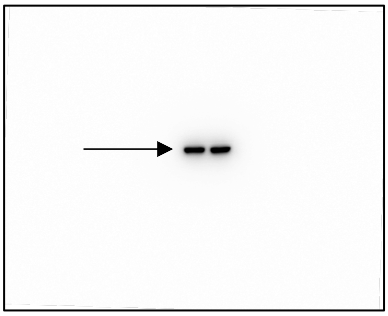

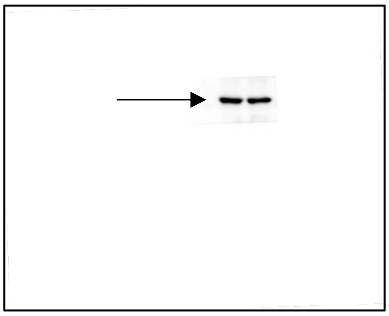

α-tubulin(Huh7) α-tubulin(LM3)

Full and uncropped western blot for Figure 2G

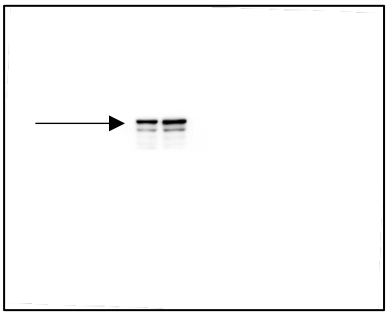

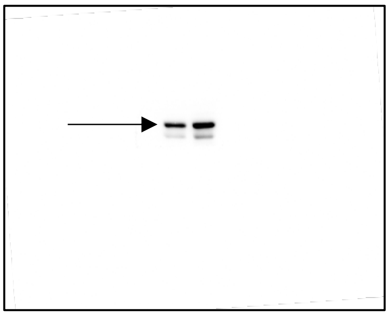

NAP1L1(Huh7) NAP1L1(LM3)

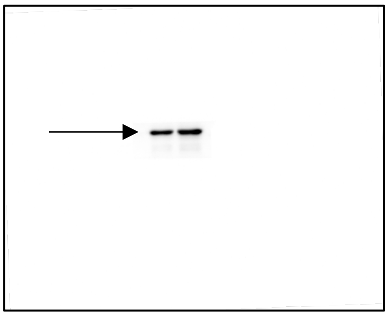

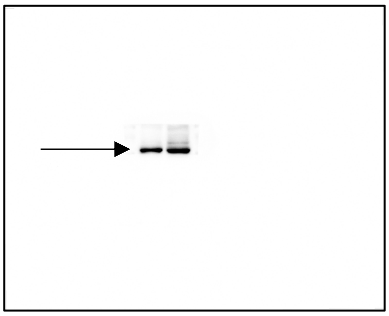

BIRC2(Huh7) BIRC2(LM3)

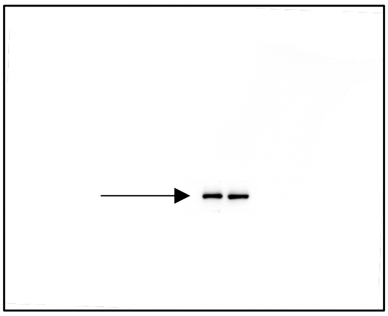

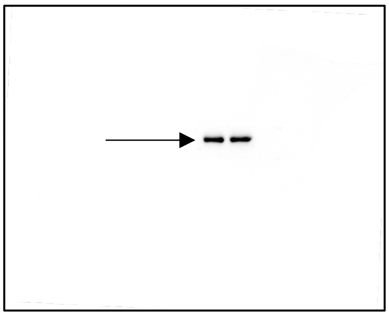

α-tubulin(Huh7) α-tubulin(LM3)

Full and uncropped western blot for Figure 2H

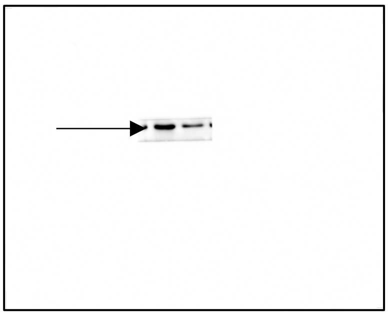

BIRC2(Huh7)

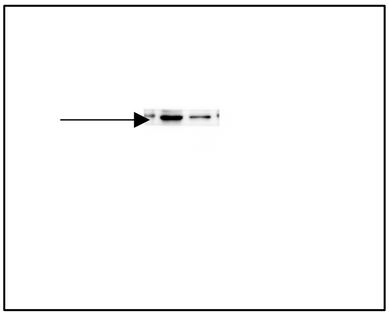

BIRC2(LM3)

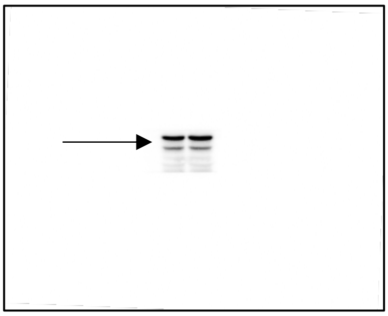

NAP1L1(Huh7)

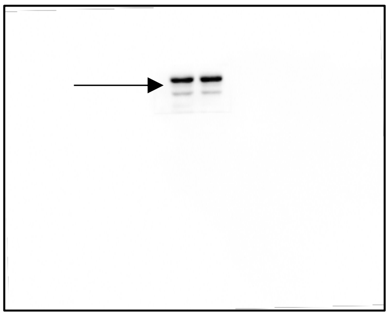

NAP1L1(LM3)

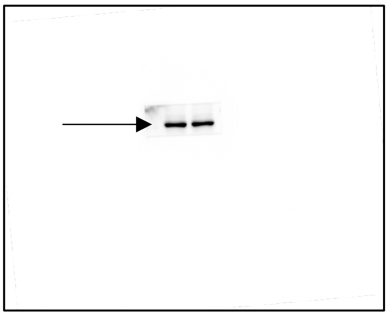

α-tubulin(Huh7)

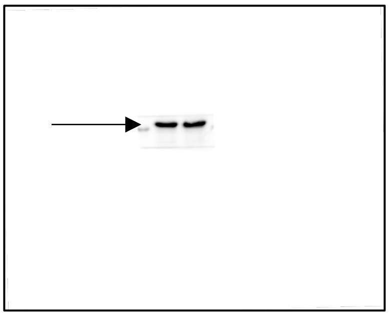

α-tubulin(LM3)

Full and uncropped western blot for Figure 2I

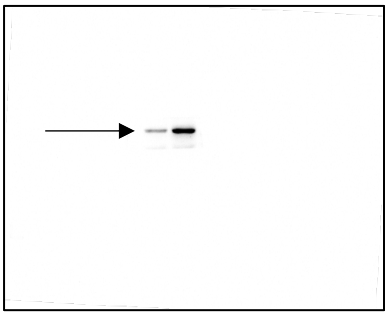

BIRC2(Huh7)

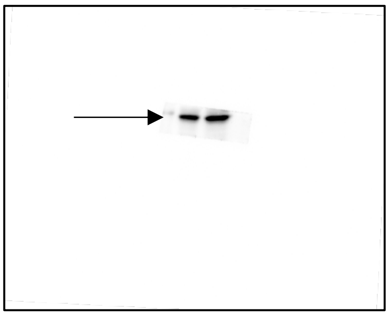

BIRC2(LM3)

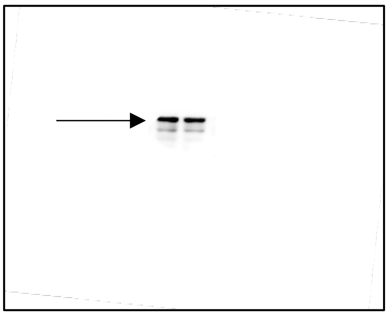

NAP1L1(Huh7)

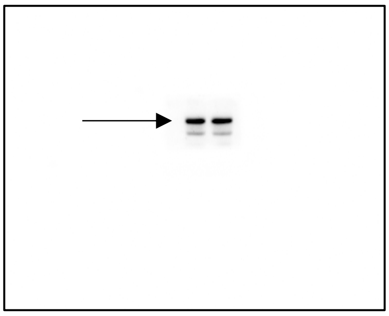

NAP1L1(LM3)

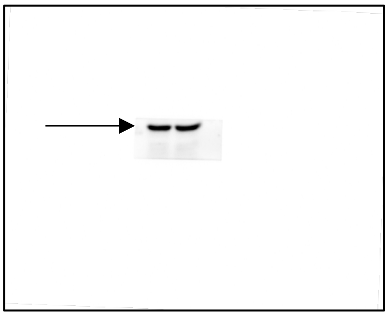
α-tubulin(Huh7)

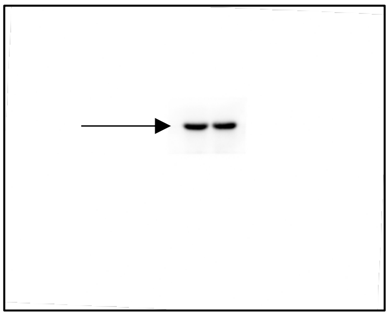
α-tubulin(LM3)

Full and uncropped western blot for Figure 4G

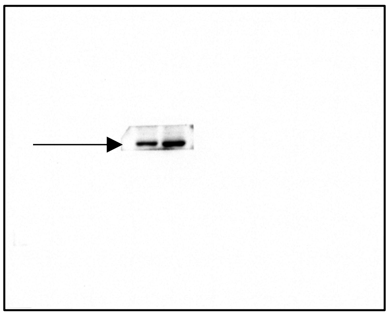
BIRC2(Huh7)

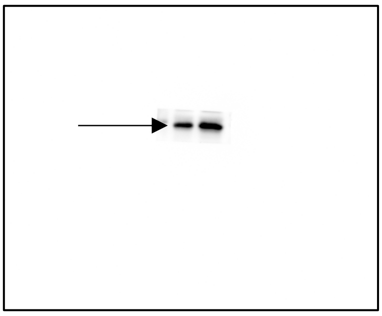
BIRC2(LM3)

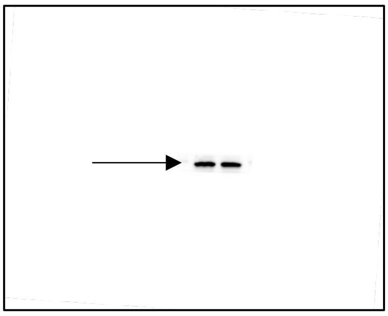
Caspase9(Huh7)

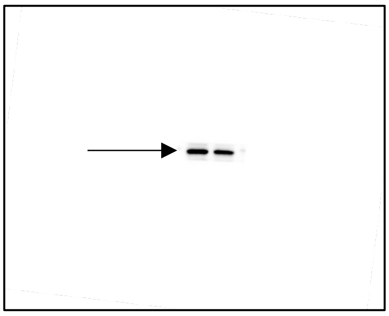
Caspase9(LM3)

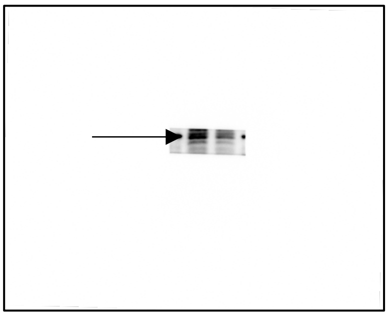
Cleaved caspase9(Huh7)

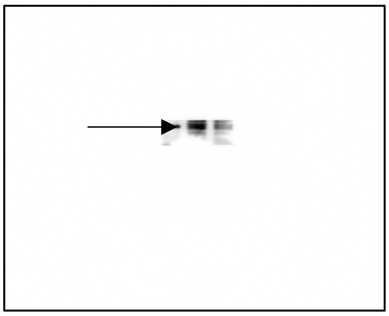
Cleaved caspase9(LM3)

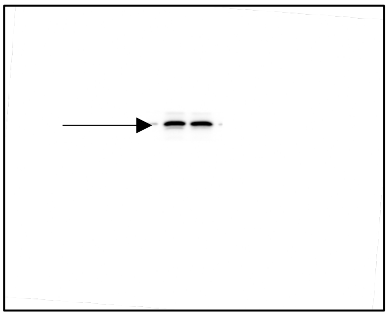
Caspase7(Huh7)

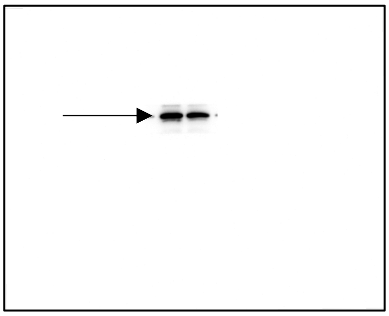
Caspase7(LM3)

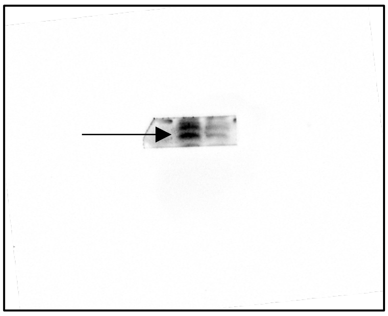

Cleaved caspase7(Huh7)

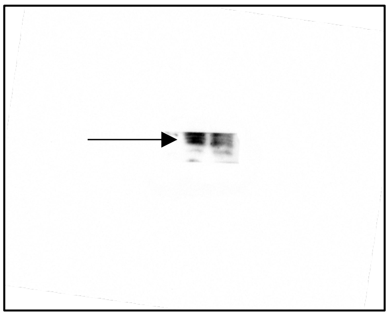

Cleaved caspase7(LM3)

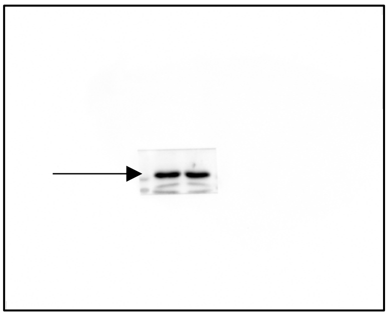

Bax(Huh7)

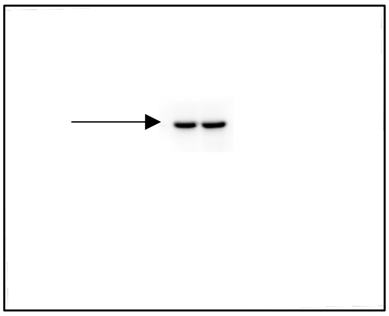

Bax(LM3)

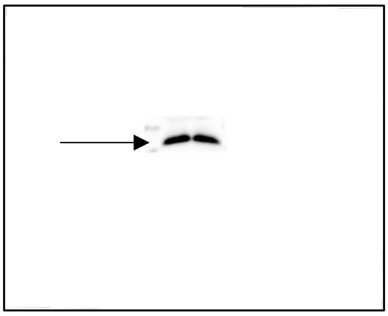

Cyt-c(Huh7)

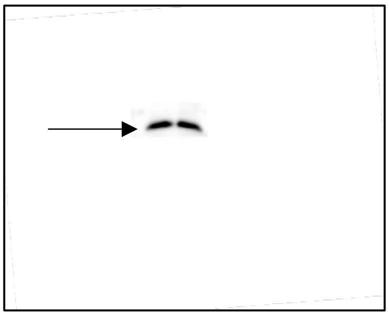

Cyt-c(LM3)

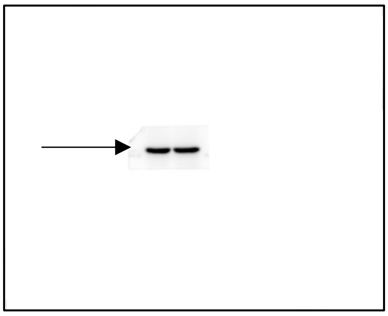

α-tubulin(Huh7)

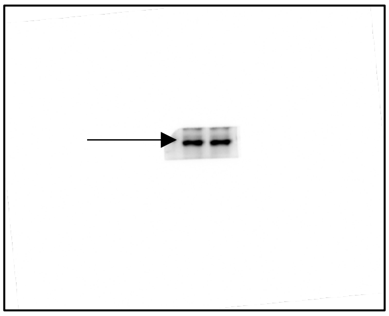

α-tubulin(LM3)

Full and uncropped western blot for Figure 4H

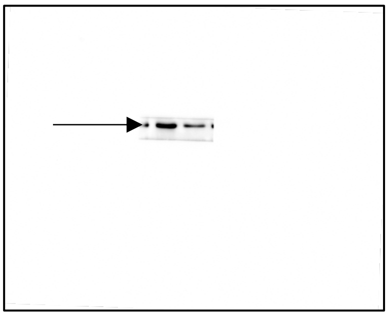

BIRC2(Huh7)

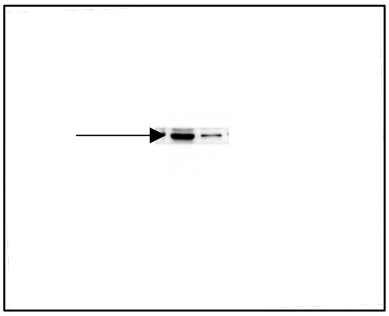

BIRC2(LM3)

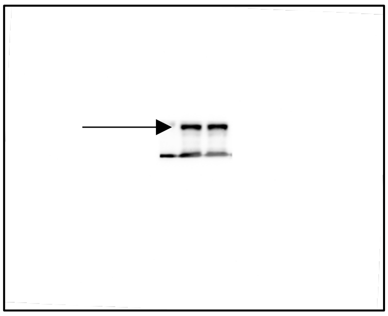
Caspase9(Huh7)

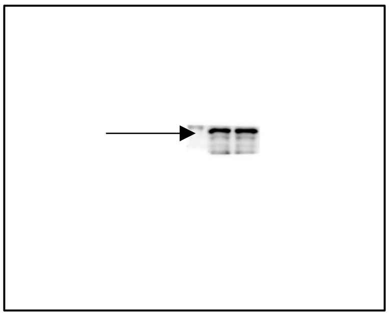
Caspase9(LM3)

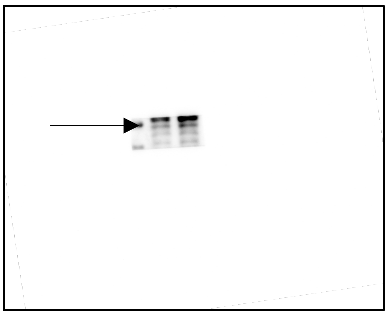
Cleaved caspase9(Huh7)

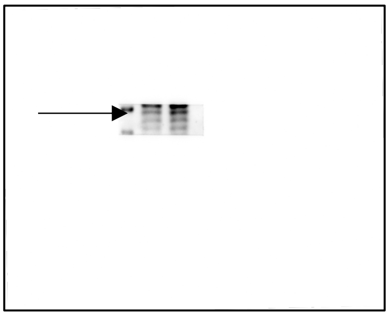
Cleaved caspase9(LM3)

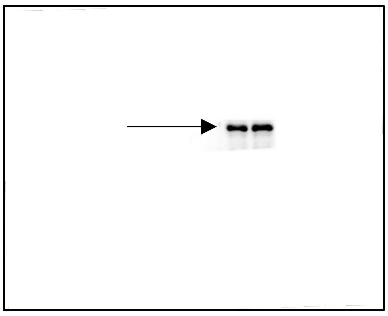
Caspase7(Huh7)

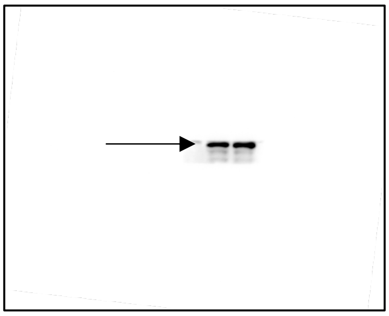
Caspase7(LM3)

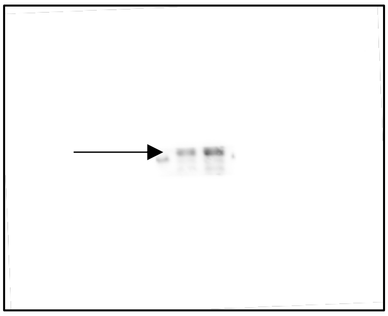
Cleaved caspase7(Huh7)

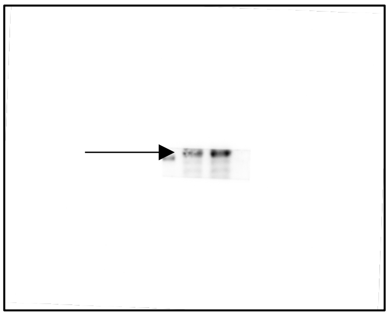
Cleaved caspase7(LM3)

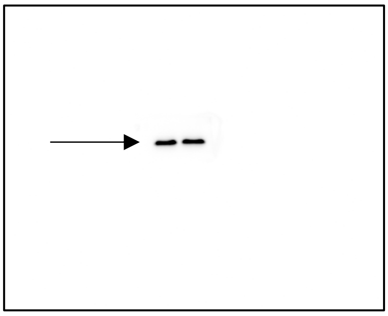
Bax(Huh7)

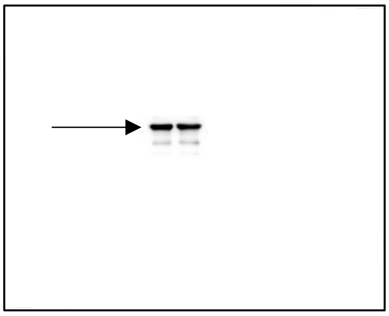
Bax(LM3)

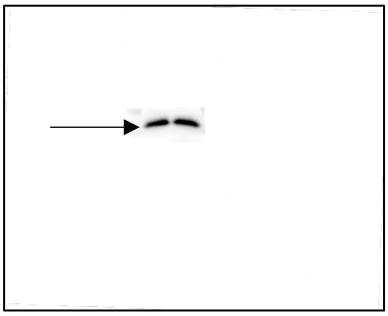

Cyt-c(Huh7)

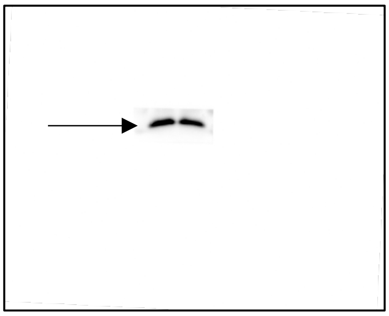

Cyt-c(LM3)

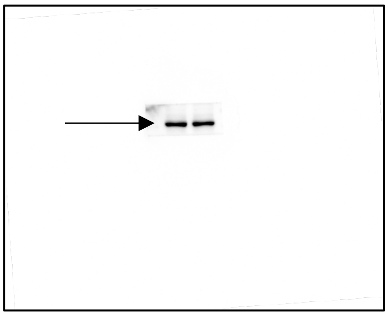

α-tubulin(Huh7)

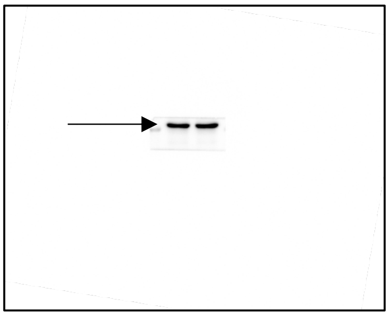

α-tubulin(LM3)

Full and uncropped western blot for Figure 5A

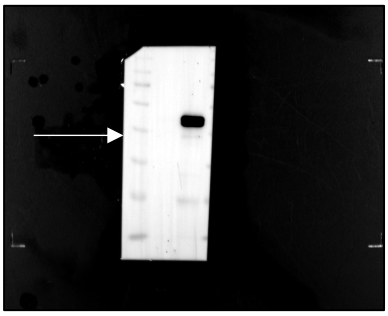

NAP1L1(IP NAP1L1)

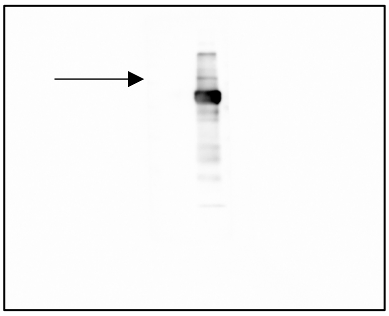

BIRC2(IP BIRC2)

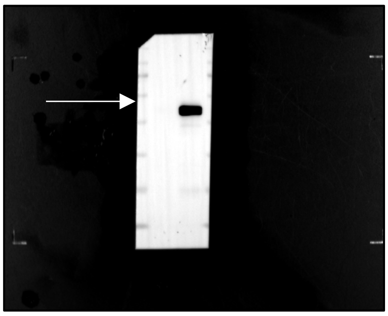

BIRC2(IP NAP1L1)

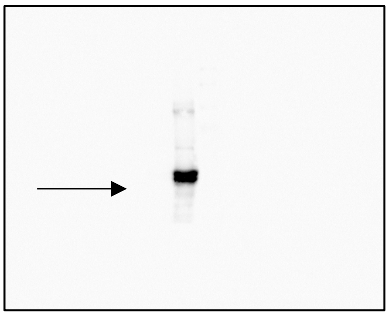

NAP1L1(IP BIRC2)

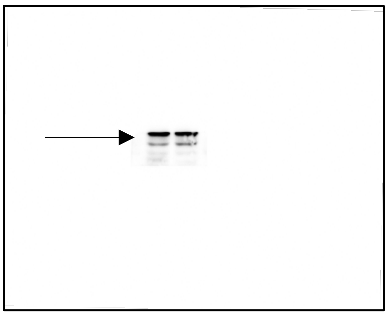

NAP1L1(input)

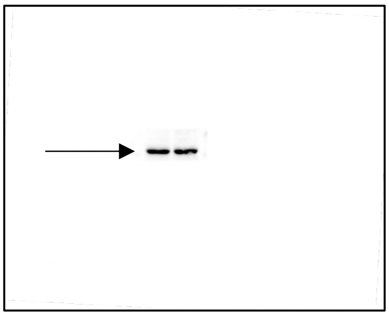

BIRC2(input)

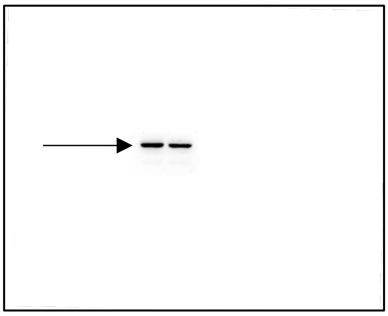

BIRC2(input)

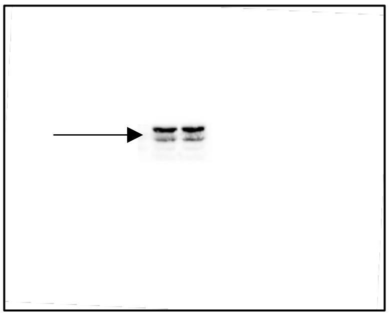

NAP1L1(input)

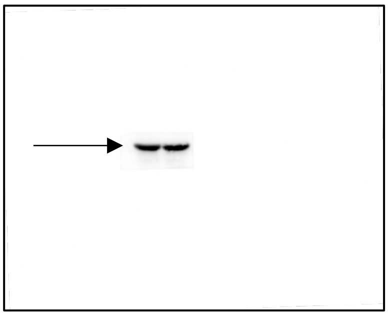

α-tubulin(input)

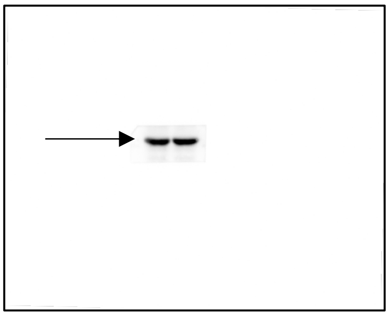

α-tubulin(input)

Full and uncropped western blot for Figure 5C

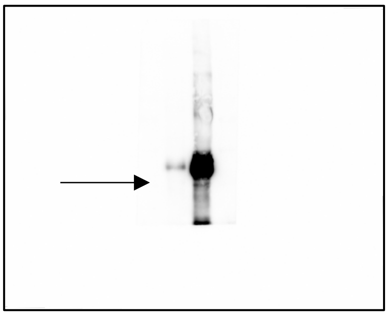

NAP1L1(IP NAP1L1)

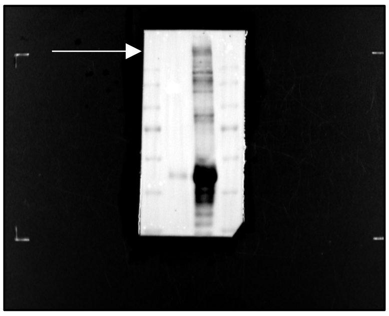

UBR4(IP UBR4)

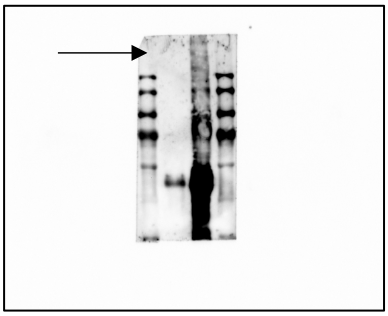

UBR4(IP NAP1L1)

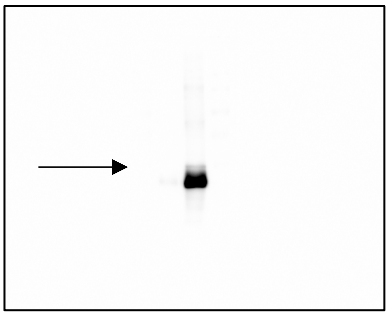

NAP1L1(IP UBR4)

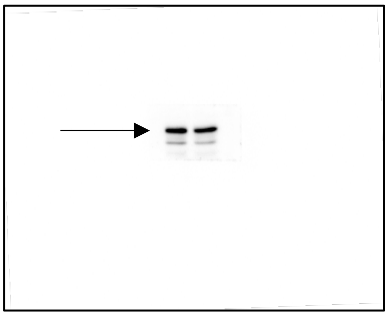

NAP1L1(input)

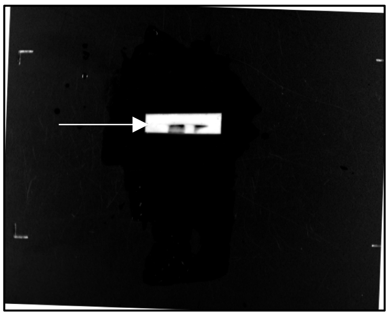

UBR4(input)

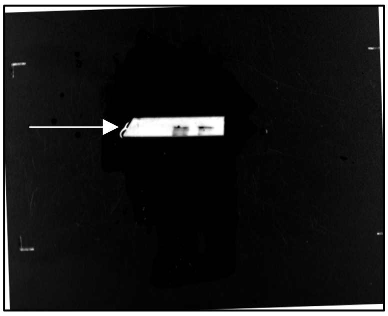

UBR4(input)

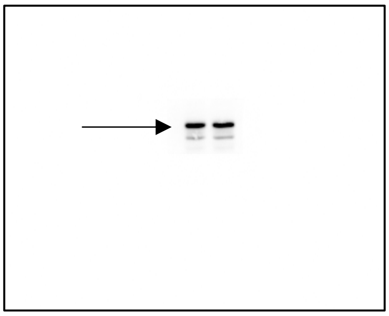

NAP1L1(input)

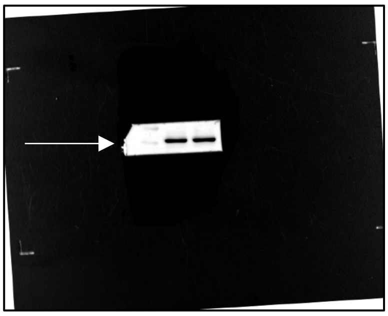

α-tubulin(input)

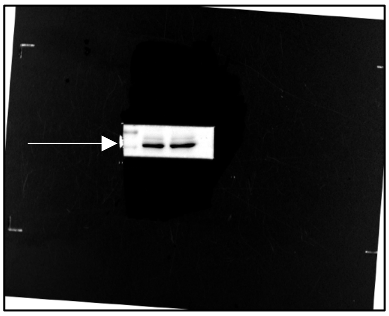

α-tubulin(input)

Full and uncropped western blot for Figure 5D

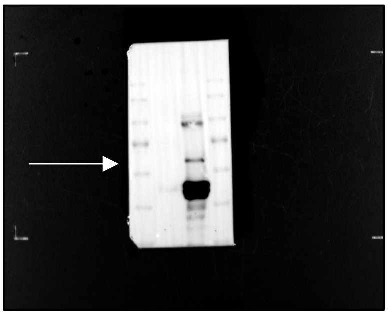

BIRC2(IP BIRC2)

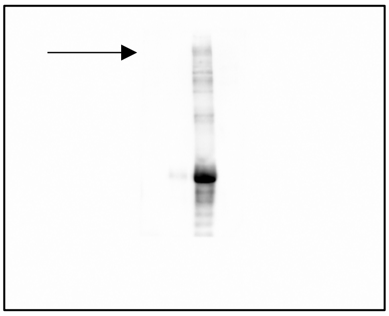

UBR4(IP UBR4)

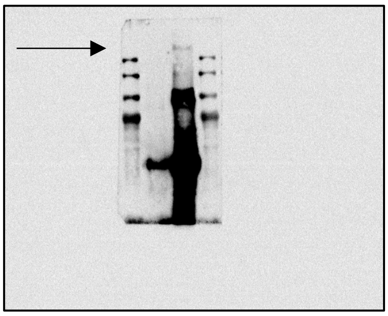

UBR4(IP BIRC2)

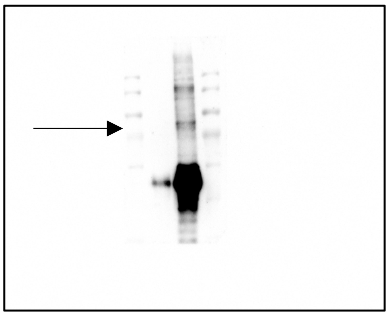

BIRC2(IP UBR4)

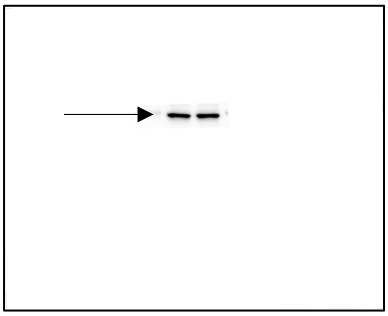

BIRC2(input)

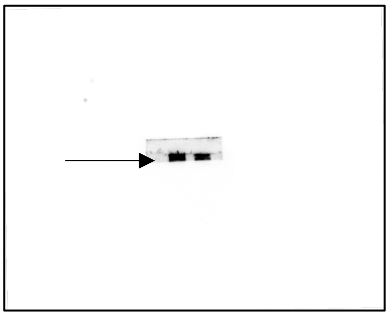

UBR4(input)

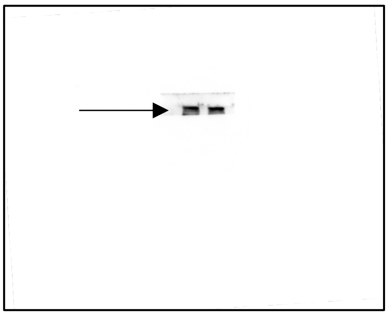

UBR4(input)

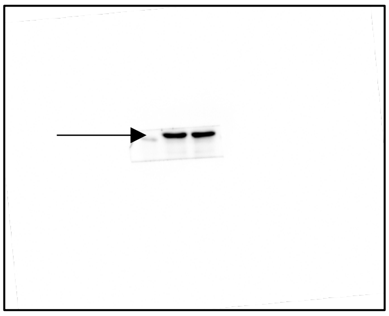

BIRC2(input)

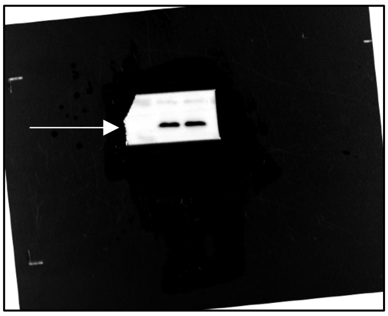

α-tubulin(input)

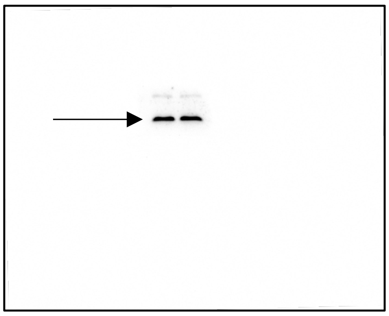

α-tubulin(input)

Full and uncropped western blot for Figure 5E

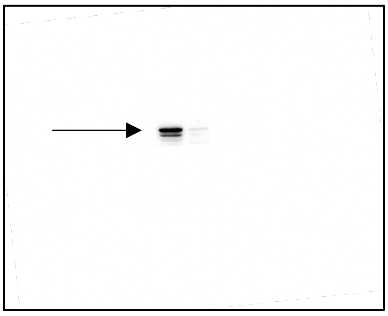

NAP1L1(Huh7)

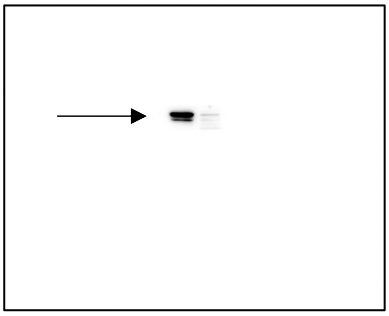

NAP1L1(LM3)

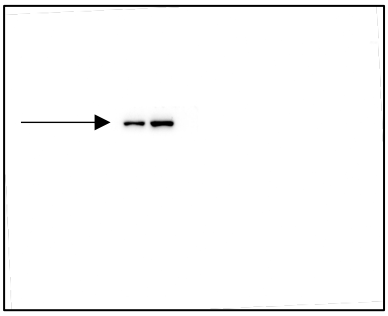

UBR4(Huh7)

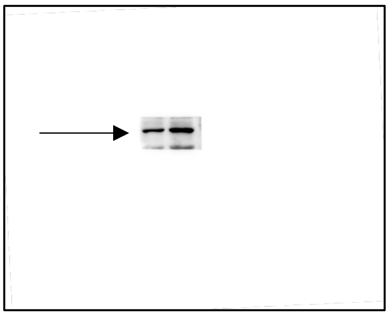

UBR4(LM3)

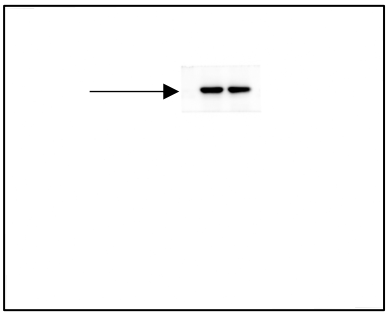

α-tubulin(Huh7)

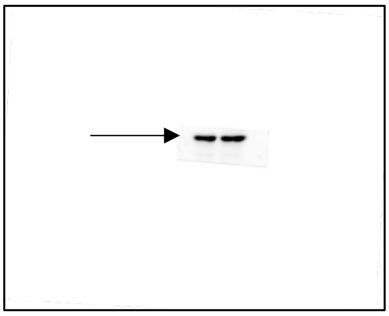

α-tubulin(LM3)

Full and uncropped western blot for Figure 5F

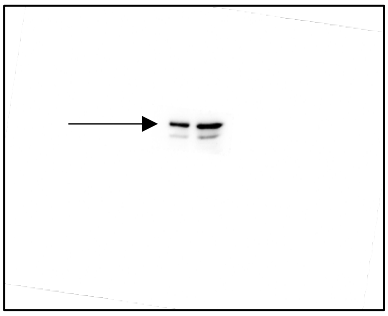

NAP1L1(Huh7)

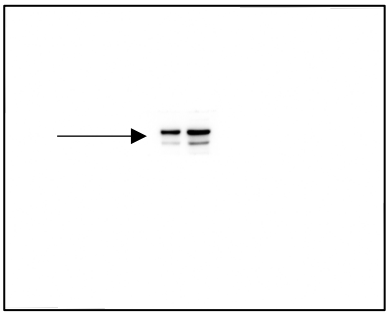

NAP1L1(LM3)

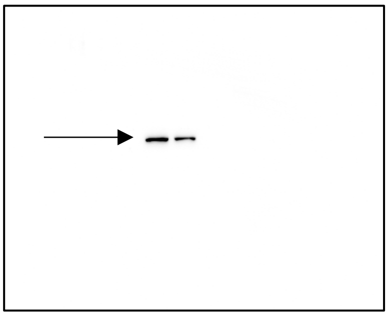

UBR4(Huh7)

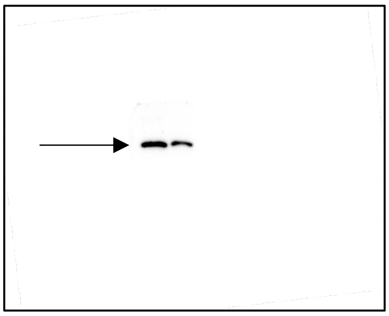

UBR4(LM3)

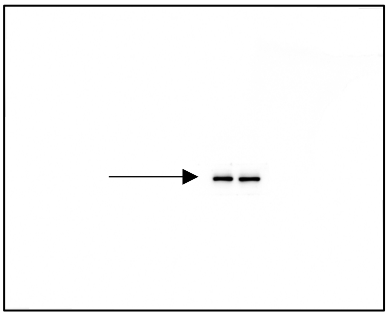

α-tubulin(Huh7)

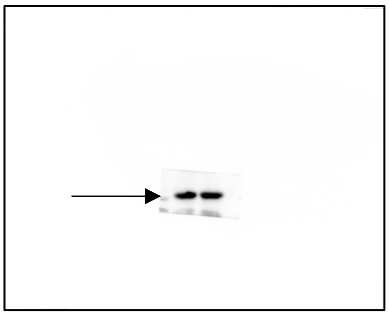

α-tubulin(LM3)

Full and uncropped western blot for Figure 5G

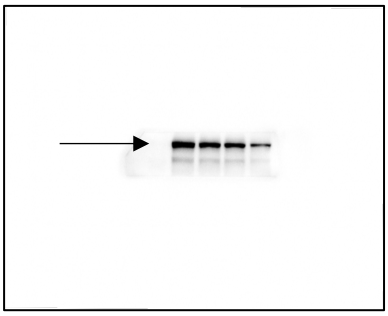

BIRC2(sh-NC)

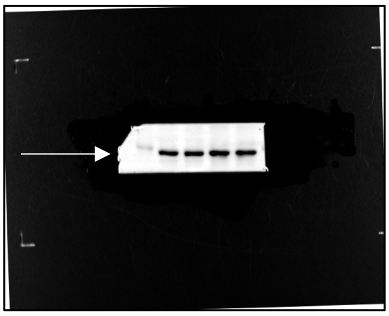

α-tubulin (sh-NC)

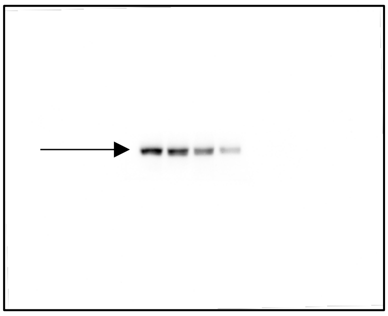

BIRC2(sh-NAP1L1)

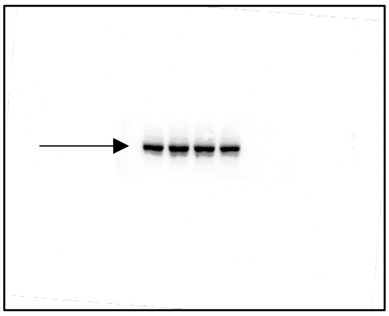

α-tubulin (sh-NAP1L1)

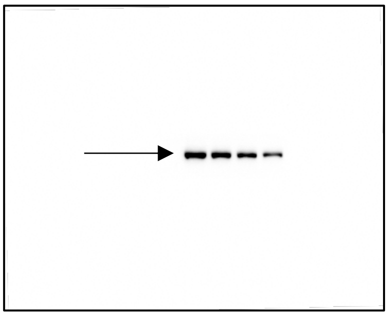

BIRC2(sh-NC)

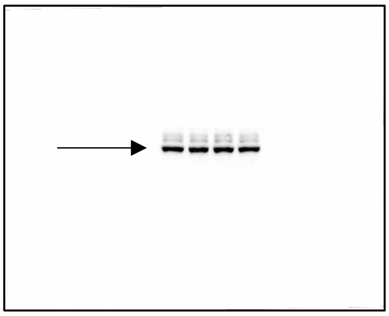

α-tubulin (sh-NC)

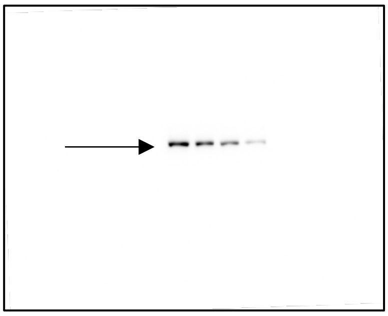

BIRC2(sh-NAP1L1)

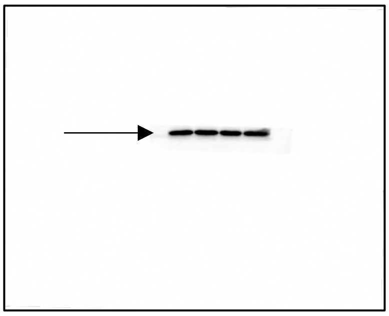

α-tubulin (sh-NAP1L1)

Full and uncropped western blot for Figure 5H

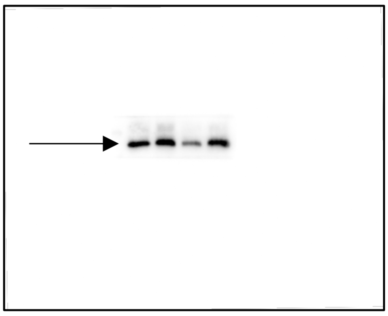

BIRC2(sh-NC)

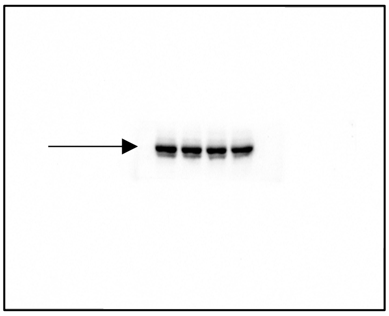

α-tubulin (sh-NC)

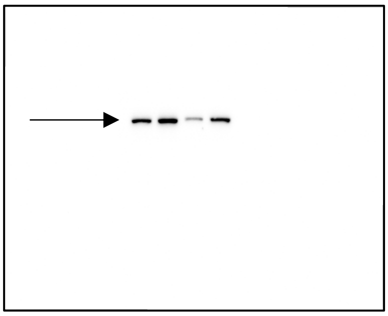

BIRC2(sh-NAP1L1)

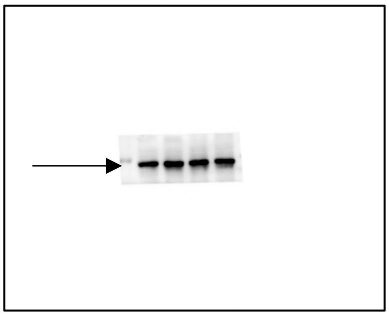

α-tubulin (sh-NAP1L1)

Full and uncropped western blot for Figure 5I

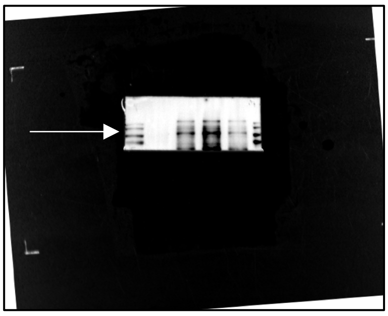

Ub(Huh7)

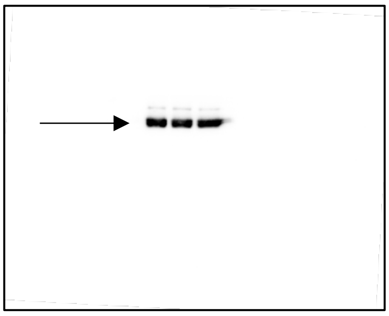

BIRC2(Huh7)

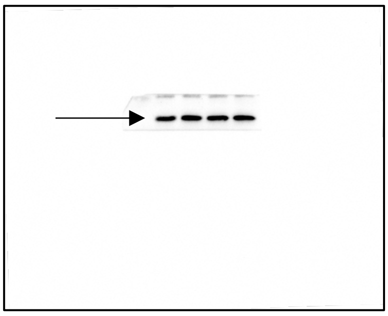

BIRC2(Huh7)

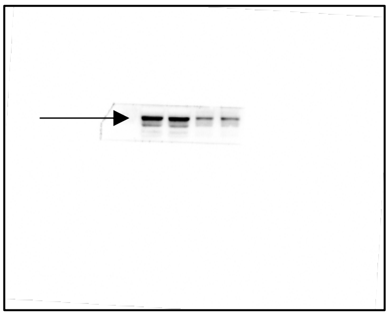

NAP1L1(Huh7)

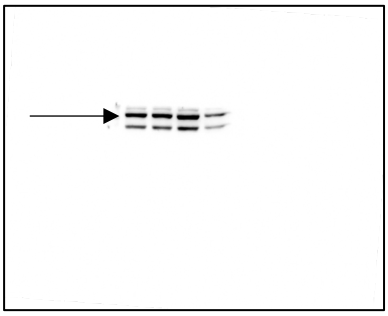

UBR4(Huh7)

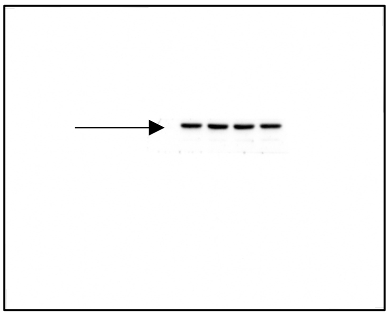

α-tubulin (Huh7)

Full and uncropped western blot for Figure 5J

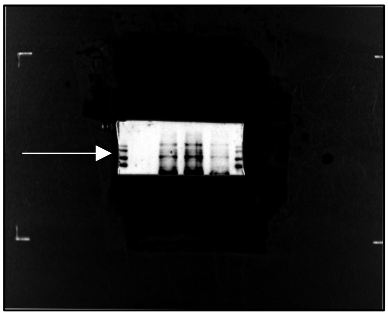

Ub(LM3)

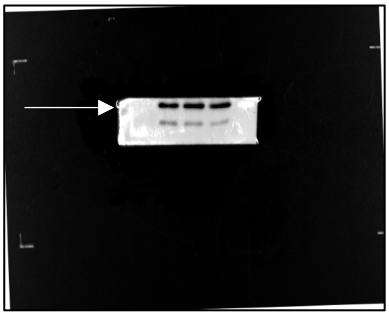

BIRC2(LM3)

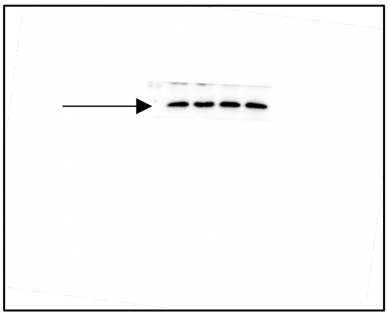

BIRC2(LM3)

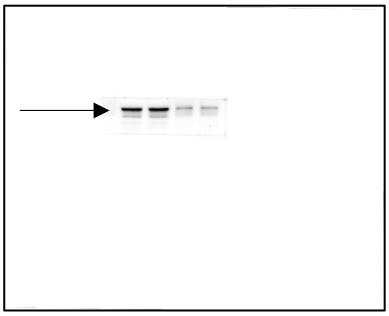

BIRC2(LM3)

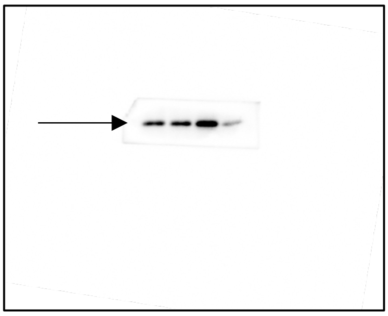

UBR4(LM3)

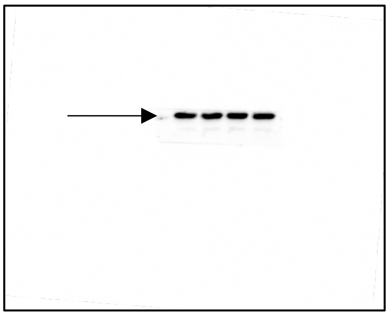

α-tubulin (LM3)

Full and uncropped western blot for Figure 6D

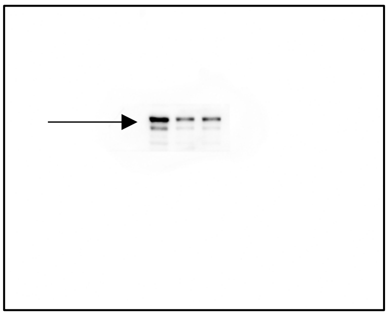

NAP1L1(Huh7)

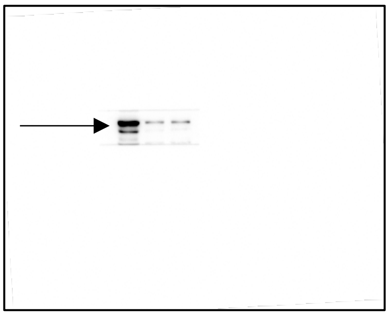

NAP1L1(LM3)

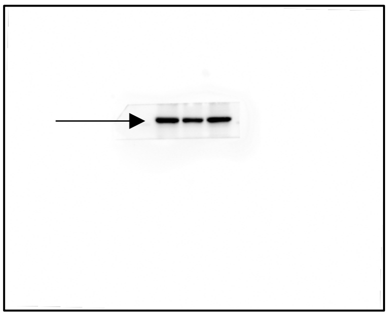

BIRC2(Huh7)

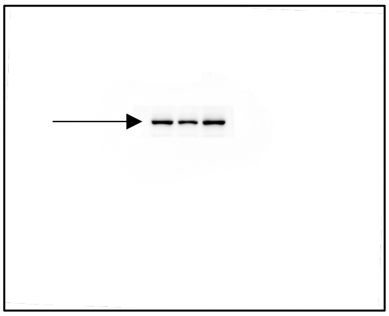

BIRC2(LM3)

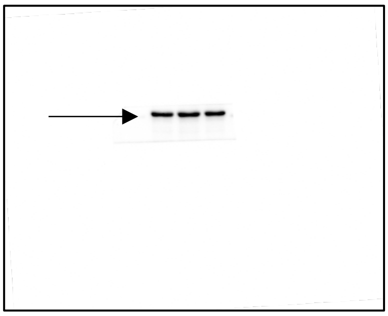

Caspase9(Huh7)

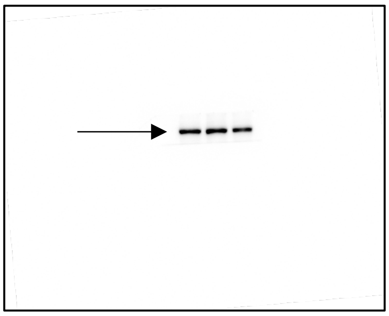

Caspase9(LM3)

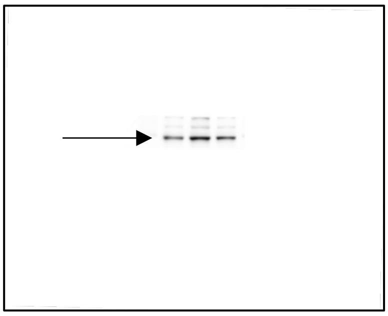

Cleaved caspase9(Huh7)

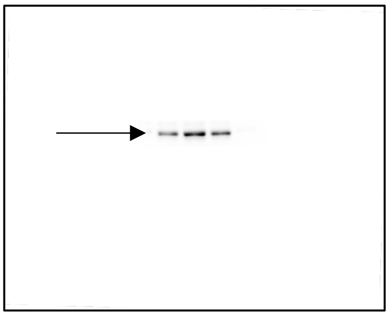

Cleaved caspase9(LM3)

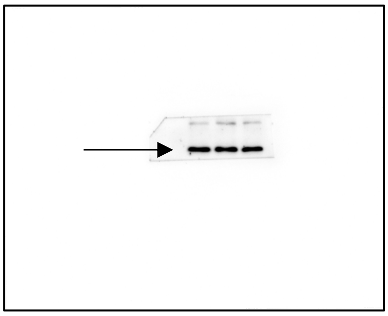

Caspase7(Huh7)

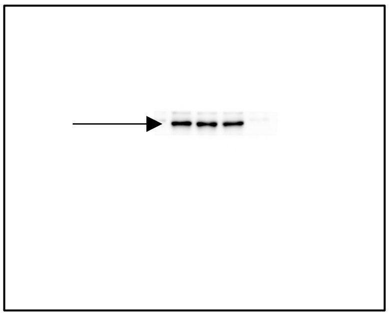

Caspase7(LM3)

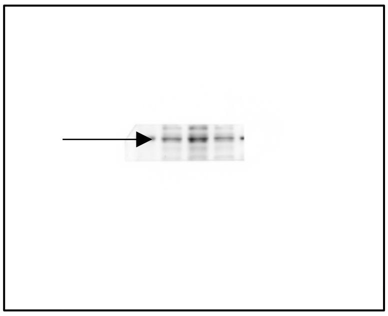

Cleaved caspase7(Huh7)

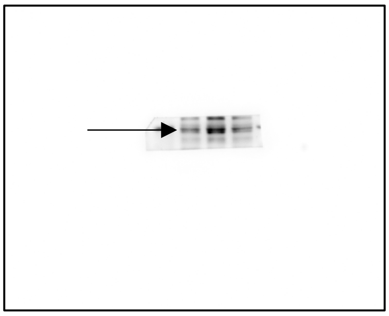

Cleaved caspase7(LM3)

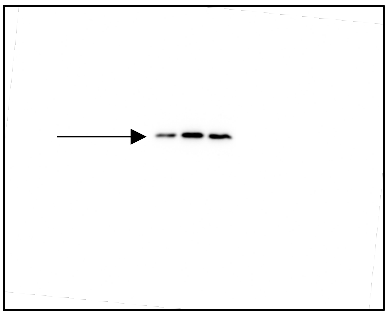

Bax(Huh7)

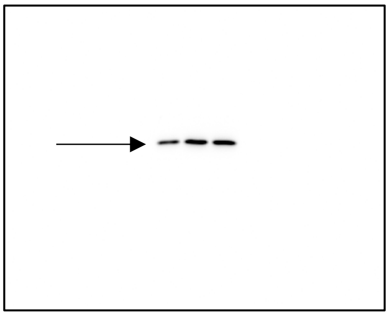

Bax(LM3)

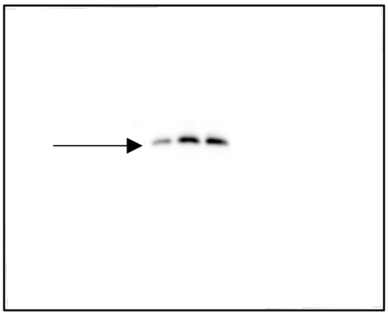

Cyt-c(Huh7)

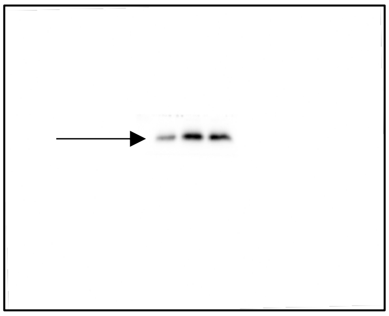

Cyt-c(LM3)

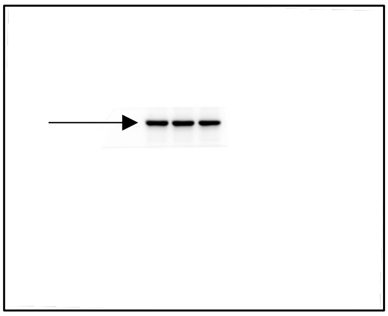

α-tubulin(Huh7)

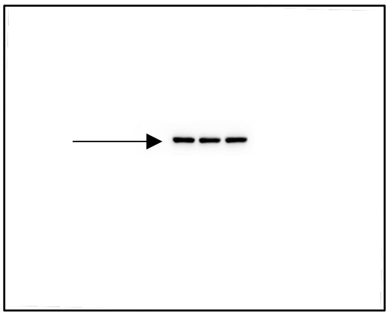

α-tubulin(LM3)

Full and uncropped western blot for Figure S1 E

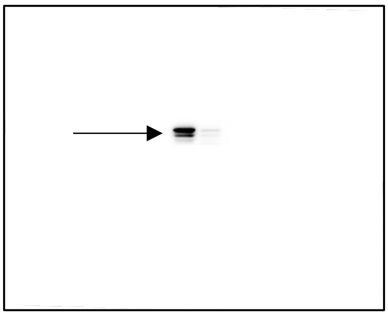

NAP1L1(Huh7)

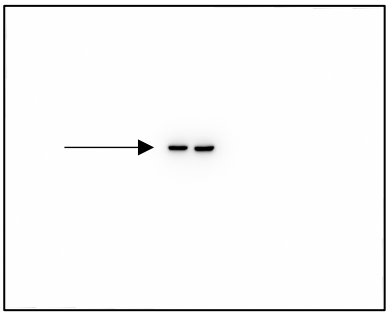

α-tubulin(Huh7)

Full and uncropped western blot for Figure S1 F

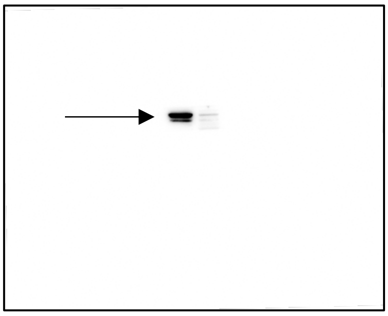

NAP1L1(LM3)

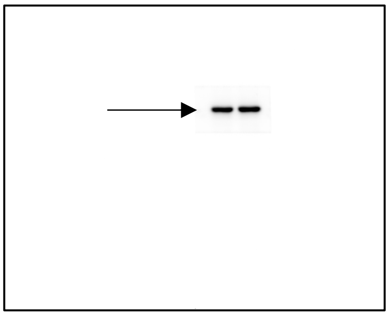

α-tubulin(LM3)

Full and uncropped western blot for Figure S1 G

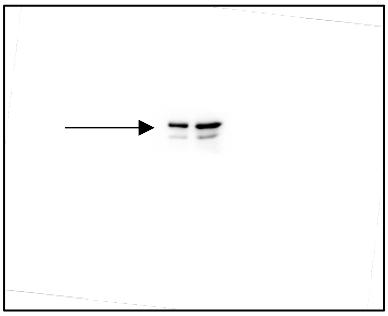

NAP1L1(Huh7)

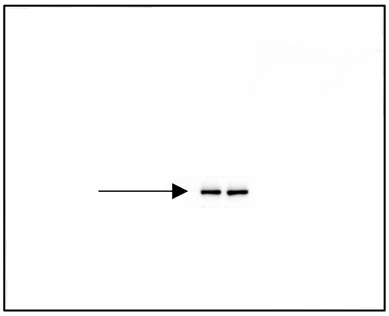

α-tubulin(Huh7)

Full and uncropped western blot for Figure S1 G

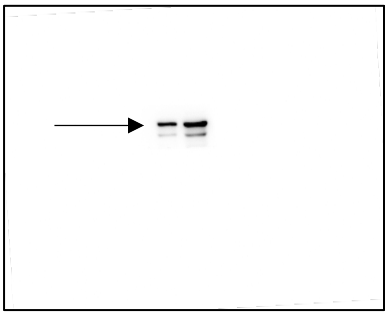

NAP1L1(LM3)

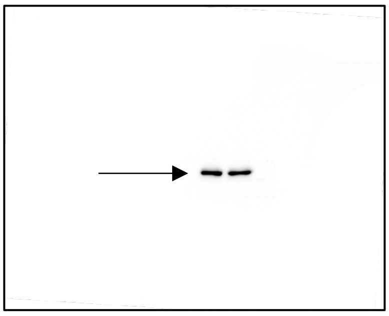

α-tubulin(LM3)

Full and uncropped western blot for Figure S2 C

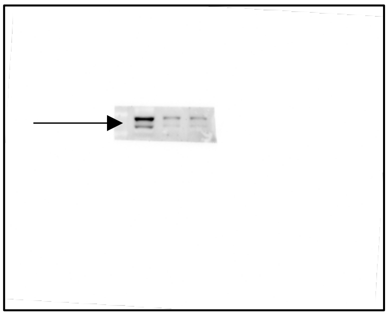

NAP1L1(Huh7)

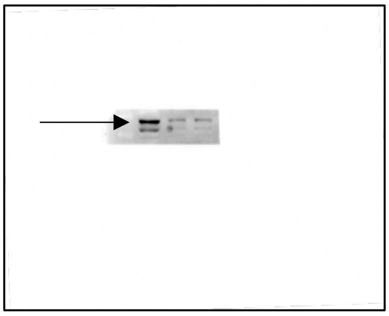

NAP1L1(LM3)

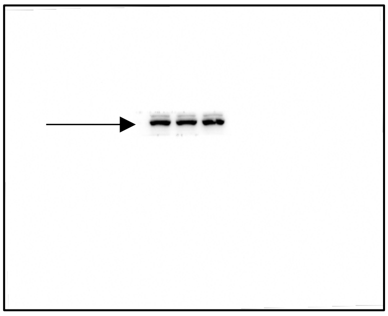

Caspase9(Huh7)

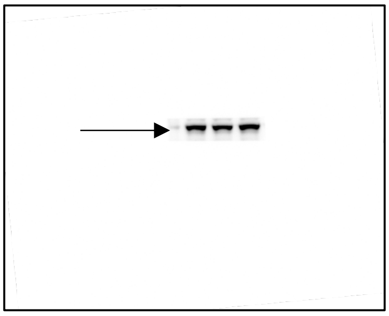

Caspase9(LM3)

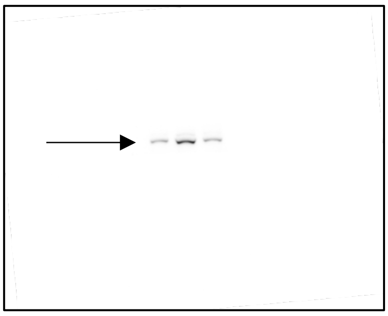

Cleaved caspase9(Huh7)

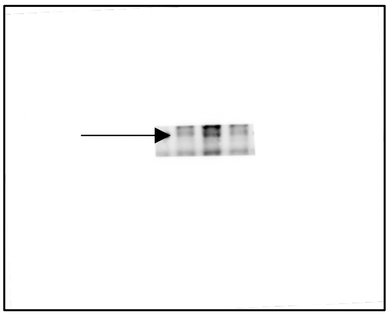

Cleaved caspase9(LM3)

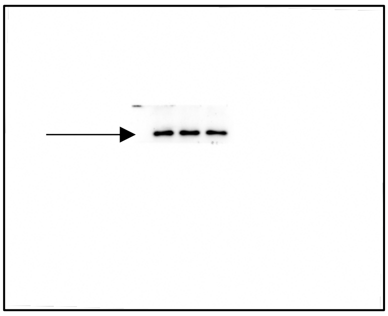

Caspase7(Huh7)

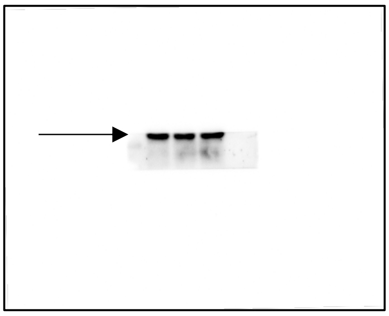

Caspase7(LM3)

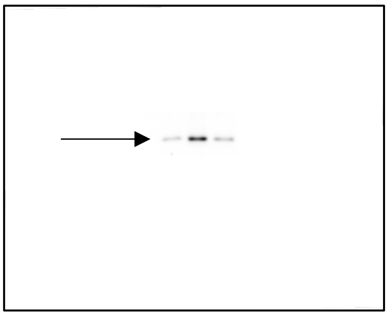

Cleaved caspase7(Huh7)

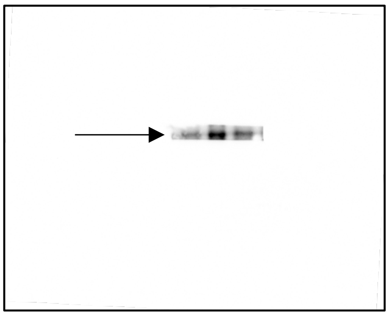

Cleaved caspase7(LM3)

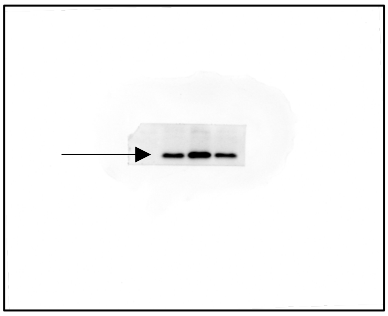

Bax(Huh7)

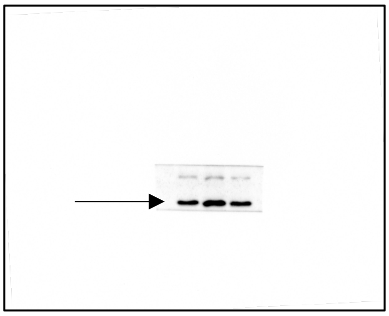

Bax(LM3)

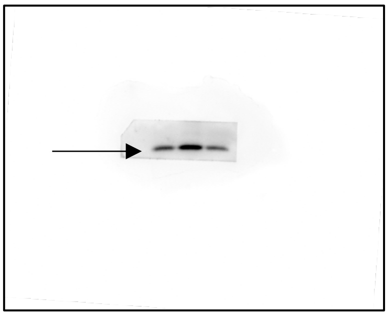

Cyt-c(Huh7)

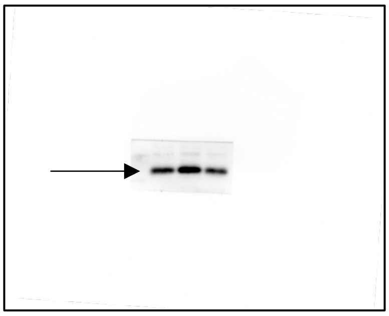

Cyt-c(LM3)

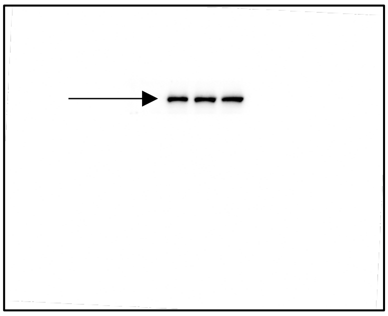

α-tubulin(Huh7)

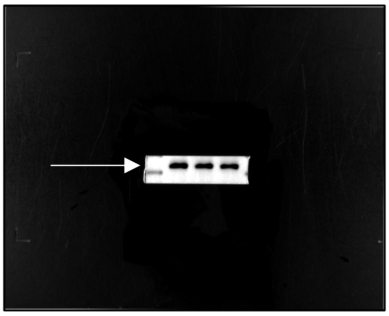

α-tubulin(LM3)

Full and uncropped western blot for Figure S4 E

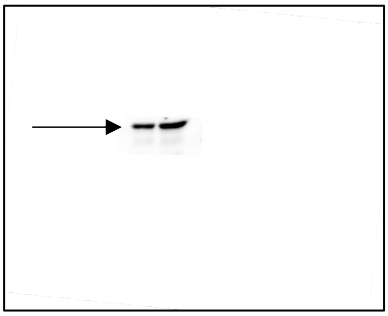

BIRC2(Huh7)

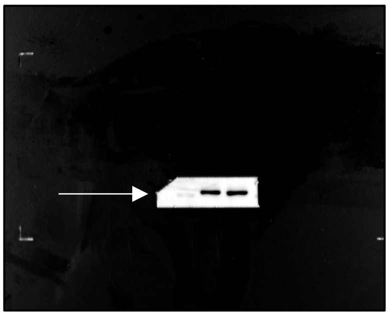

α-tubulin(Huh7)

Full and uncropped western blot for Figure S4 F

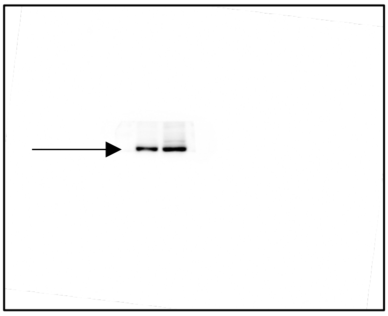

BIRC2(LM3)

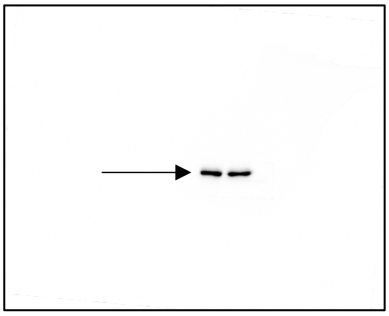

α-tubulin(LM3)

Full and uncropped western blot for Figure S4 G

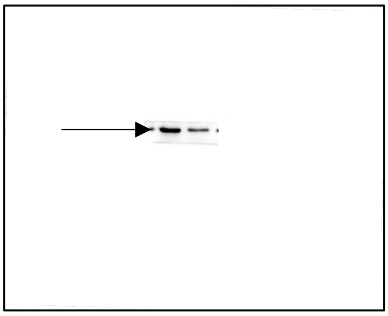

BIRC2(Huh7)

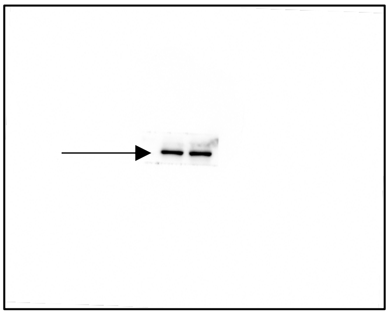

α-tubulin(Huh7)

Full and uncropped western blot for Figure S4 H

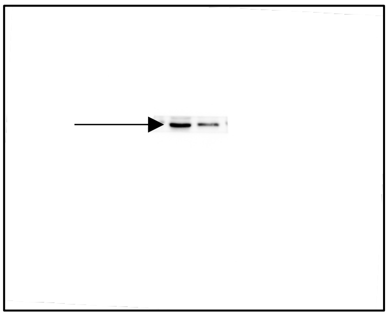

BIRC2(LM3)

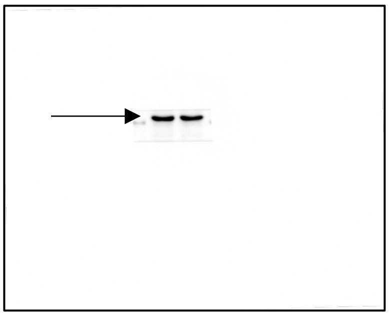

α-tubulin(LM3)

Full and uncropped western blot for Figure S4 I

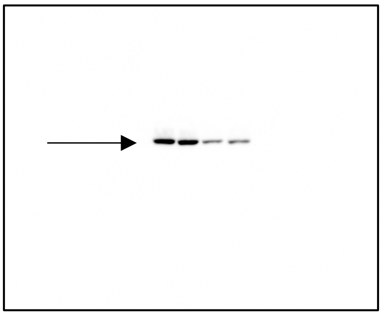

BIRC2(Huh7)

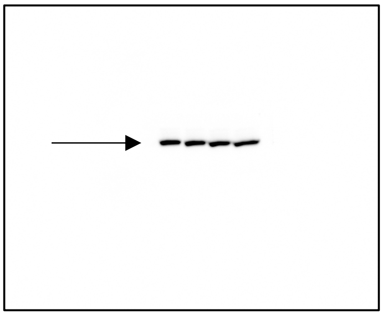

α-tubulin(Huh7)

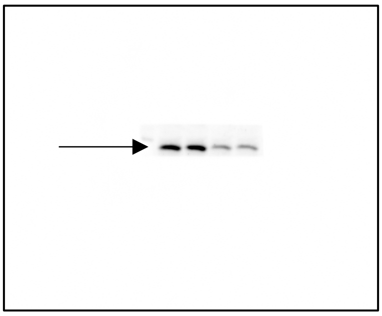

BIRC2(LM3)

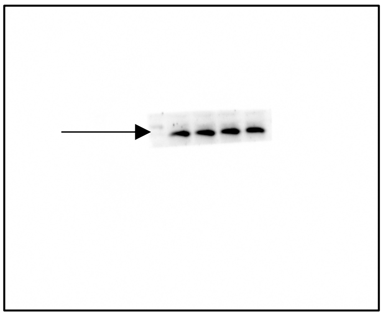

α-tubulin(LM3)

Full and uncropped western blot for Figure S4 J

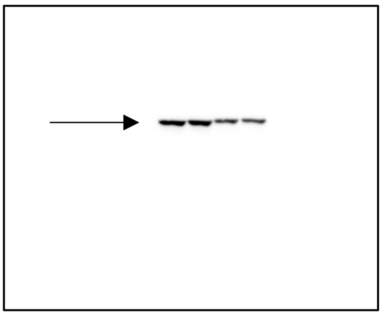

BIRC2(Huh7)

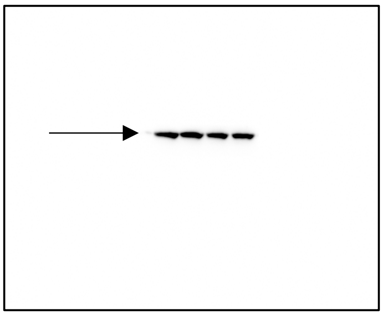

α-tubulin(Huh7)

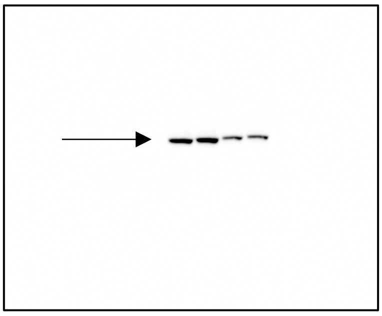

BIRC2(LM3)

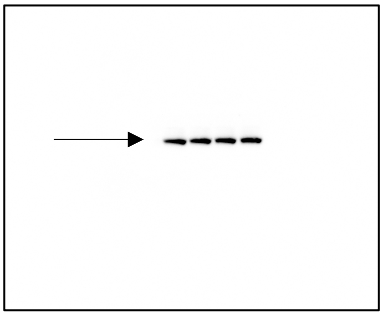

α-tubulin(LM3)
